# Harnessing the Potential of 5-Hydroxymethylfurfural: Investigating Solubility and Stability in Tailored Deep Eutectic Solvents

**DOI:** 10.1021/acssuschemeng.4c10788

**Published:** 2025-04-02

**Authors:** Grazia Isa C. Righetti, Maria Enrica Di Pietro, Gabriella Leonardi, Arianna Sinibaldi, Andrea Mezzetta, Lorenzo Guazzelli, Andrea Mele

**Affiliations:** † Department of Chemistry, Materials and Chemical Engineering “G. Natta”, Politecnico di Milano, Piazza L. da Vinci 32, 20133 Milano, Italy; ‡ Dipartimento di Farmacia, Università di Pisa, Via Bonanno 6, 56126 Pisa, Italy

**Keywords:** biomass, HMF, DES, sustainability, green chemistry, EcoScale, solubility, stability

## Abstract

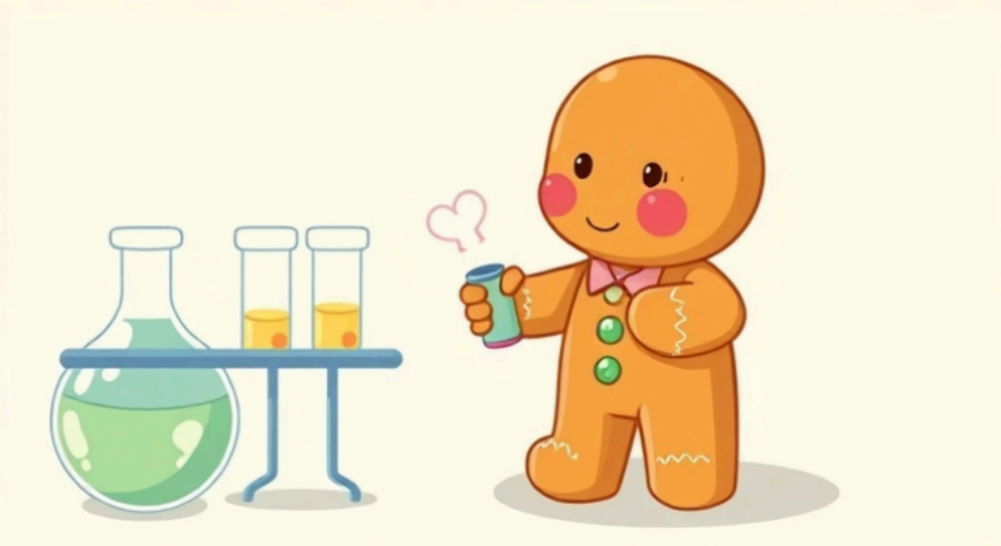

The potential of 5-hydroxymethylfurfural (HMF) as a platform chemical for sustainable biobased products is widely recognized. However, its practical applications are often constrained by stability challenges. This study examines the solubility and chemical stability of HMF in 15 paradigmatic deep eutectic solvents (DES), formed by combining five hydrogen bond acceptors (HBAs: betaine, choline chloride, choline acetate, L-carnitine, and l-proline) and three hydrogen bond donors (HBDs: glycerol, ethylene glycol, and levulinic acid). Our findings reveal that HMF demonstrates high solubility and remarkable stability across the studied DES. Only a few cases of HMF degradation were observed and discussed, with specific degradation pathways identified in certain solvent mixtures. Notably, the HBAs within the DES play a crucial role in significantly enhancing the stability of HMF, establishing a foundation for its use as a renewable synthon in organic chemistry using environmentally benign solvents. These findings represent a significant step forward in aligning the synthetic design with the principles of green chemistry.

## Abstract 2

The solubility and chemical stability of 5-hydroxymethylfurfural (HMF) was studied in 15 representative DES.

## Introduction

The synthesis of chemicals from renewable resources represents a pivotal advancement in addressing the multifaceted environmental crisis that our planet is currently facing. Traditional chemical synthesis was predominantly reliant on petrochemical feedstocks, whose extraction and utilization have led to severe environmental repercussions, including greenhouse gas emissions, pollution, and resource depletion. As the global community copes with the escalating challenges of climate change, biodiversity loss, and the unsustainable exploitation of natural resources, developing sustainable and eco-friendly chemical processes has become imperative. Renewable resources, such as biomass, offer a viable and sustainable alternative to conventional fossil fuels, and during the past decade, there has been an exponential increase in the interest in synthesizing chemicals from natural sources to reduce the reliance on petroleum derivatives.^1−5^ The rising demand for chemicals from renewable sources such as biomass has placed it at the forefront of research. In this context, lignocellulosic biomass is an important source of carbohydrates, and its refining allows for the preparation of many platform molecules, actually contributing to approaching the 21st century sustainability target of “Replacing all petroleum-based chemical products with natural biomass-based chemical products”.^6^

Among all, 5-hydroxymethylfurfural (HMF) has been classified as one of the most promising cellulose-derived platform chemicals for the synthesis of high-value-added molecules and high-performance polymers thanks to the presence of the rigid structure of the furan ring.^7,8^ The dehydration of monosaccharides and polysaccharides, which are abundant in biomass, represents the primary synthesis route for HMF production. Cellulose can be transformed into HMF through three steps: (i) the hydrolysis of β-(1→4) glycosidic bonds to obtain glucose units, followed by (ii) the isomerization of glucose into fructose, and finally (iii) the acid-catalyzed dehydration of this latter.^9−15^ HMF presents a peculiar chemical structure that combines the aromaticity of the furan ring with hydroxyl and aldehyde functional groups (FGs). The presence of different FGs allows HMF transformation in a variety of value-added molecules such as 2,5-dimethyl furane (DMF),^15^ 5-hydroxymethyl-2-furancarboxylic acid (HMFCA), 2,5-diformylfuran (DFF), 5-formyl-2-furancarboxylic acid (FFCA), and 2,5-furan dicarboxylic acid (FDCA).^9,16,17^ The biobased nature, together with the presence of different FGs and the low toxicity, are the features that make HMF highly attractive. However, this molecule is characterized by low stability, such that the presence of even traces of impurities might favor the formation of its dimer or oligomer.^18−20^ This makes HMF difficult to produce and purify, increasing costs and limiting industrial use. For these reasons, HMF is considered the “sleeping giant” of renewable building blocks. Very recently, Ananikov and co-workers^21^ reported the stability of HMF and other related furanic compounds in a set of traditional organic solvents. They showed that subtle interplaying effects occur and that understanding and profiling solvent versus stability is at the base of the full exploitation of biobased platform chemicals.

In the current era of green and sustainable chemistry, deep eutectic solvents (DESs) have gained much attention as a new and innovative alternative to the commonly employed reaction media. Deep eutectic systems are a neoteric class of solvent strongly related to ionic liquids (IL) with which they share some general characteristics as high thermal stability and low volatility.^22^ They derive from the combination of two or more components whose interaction gives rise to an eutectic mixture characterized by a melting point depression larger than that of the ideal eutectic.^23^ The concept of deep eutectic mixture was first introduced in the scientific literature by Abbott and co-workers in 2003,^24,25^ and since then, they have been widely studied. Typically, DESs are made by mixing compounds with hydrogen bond donor (HBD) and hydrogen bond acceptor (HBA) characteristics. DESs precursors are often derived from natural and renewable sources, are usually biodegradable, and exhibit low toxicity. Additionally, DESs are commonly prepared by mixing the pure components, thus with 100% atom economy and zero waste. These attributes, along with often favorable market availability and low cost of the individual components, make DESs an attractive alternative to traditional organic solvents, which often suffer from toxicity and environmental impact due to high vapor pressure, and are mostly derived from nonrenewable petroleum resources. Interestingly, the properties of DESs can be tuned by varying both the nature and the ratio of the HBD and HBA components. This tunability allows for the design of solvents with specific characteristics tailored to particular applications, ranging from chemical synthesis to gas capture,^26^ biomass processing,^27−30^ drug delivery, and pharmaceutical formulations. Their unique properties are often likely to position DES within the intersection of environmental, economic, and social requirements for sustainability, thus accomplishing the modern definition of sustainable products.^31^ This makes them a crucial component of sustainable development in various industrial and research applications by providing a safer, greener alternative to traditional solvents.^32^

Given the potential of both HMF and DESs toward sustainable development, this work aims to fill a critical gap in the current understanding of HMF stability and solubility within DES systems, providing valuable experimental data. The chemical stability of HMF in selected DES is a necessary, although not sufficient, condition for the rational development of new synthetic approaches based on the aforementioned principles. In this work, we monitor the response of HMF to DES under temperature and residual water content conditions that are representative of a common and appropriate initial set of experimental parameters, serving as a reference benchmark for future advancements. The same rationale applies to the selection of DES, which we intentionally chose among the most common ones to ensure the generality and broad applicability of our findings. Given that the primary objective of our work is to demonstrate the feasibility of HMF-based chemistry in DES as a practical foundation for a novel, nonpetroleum-based approach to organic synthesis, this choice is both justified and essential. Thus, through spectroscopic monitoring, we assess the chemical stability of 5-hydroxymethylfurfural (HMF) in paradigmatic deep eutectic solvents (DES). By elucidating these fundamental properties, we lay the foundation for future research on the transformation and valorization of HMF in DES systems. Our findings could significantly contribute to the development of sustainable and efficient processes in green chemistry, opening new avenues for HMF applications in diverse chemical transformations within DES media.

## Results and Discussion

Fifteen hydrophilic model deep eutectic solvents (DESs) were prepared and tested, as summarized in Table 1.

**Table 1:** Water Content of the DES Systems over Time, Stability, and EcoScale Analysis

DES	physical appearance	water content (wt %) - *t*_0_^a^	water uptake (wt %)^b^	stability	EcoScale
Bet-Gly	homogeneous, colorless	0.5	10.2	S	98
Bet-LA	homogeneous, colorless	0.6	4.9	S	93
Bet-EG	homogeneous, colorless	0.5	12.0	S	93
ChCl-Gly	homogeneous, colorless	0.6	10.3	S	98
ChCl-LA	homogeneous, colorless	0.6	11.6	S	93
ChCl-EG	homogeneous, colorless	0.8	16.3	S	93
ChAc-Gly	homogeneous, colorless	0.6	14.5	S	98
ChAc-LA	homogeneous, colorless	0.6	11.0	S	93
ChAc-EG	homogeneous, colorless	1.6	16.6	S	93
Carn-Gly	homogeneous, colorless	0.6	9.5	S	95
Carn-LA	homogeneous, colorless	0.6	4.5	S	90
Carn-EG	homogeneous, colorless	0.8	15.2	S	90
Pro-Gly	homogeneous, light yellow	0.6	6.9	S	98
Pro-LA	homogeneous, light yellow	0.6	4.6	S	93
Pro-EG	heterogeneous, light yellow	0.8	NG^c^	NS	93

a *t*_0_ = water content of the freshly prepared mixture.

b Water uptake of the same sample after 2 weeks.

c NG = not given due to heterogeneity of the sample; S = stable; NS = not stable (two-phase system when brought back at room temperature).

**Figure 1:**
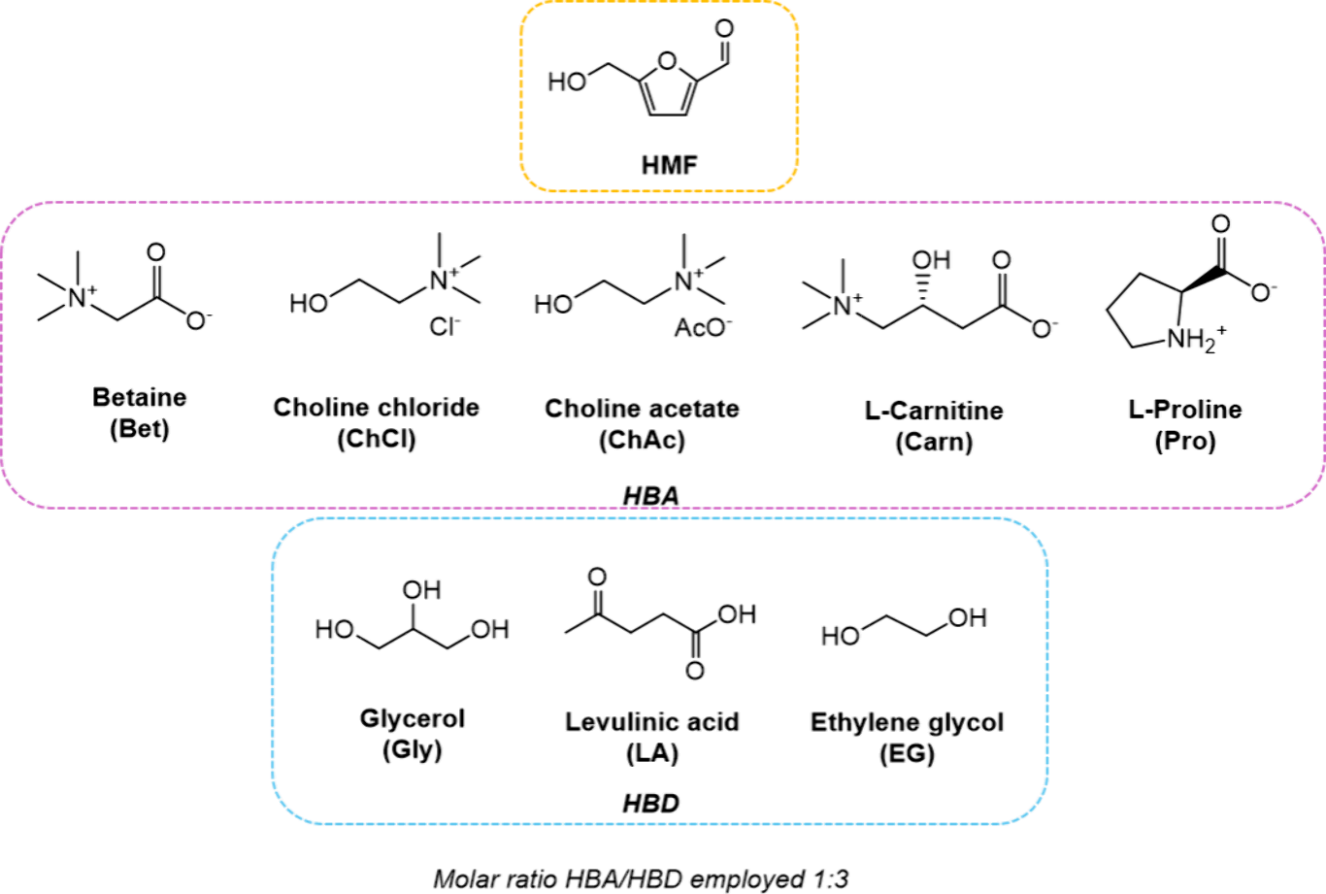
Schematic representation and chemical structure of all the compounds used in this work.

The molecular structures of the selected compounds are shown in Figure 1, including 5-hydroxymethylfurfural (HMF), the hydrogen bond acceptors (HBAs, here indicated with abbreviations: Bet, ChCl, ChAc, Carn, and Pro), and the hydrogen bond donors (HBDs, Gly, LA, and EG). The previous literature on DESs already highlighted their high thermal stability^33^ and reported on their volatility^34^; therefore, this work investigated the physicochemical properties and long-term chemical stability of these DESs, with a focus on parameters such as water content, water absorption from air over time, density, and viscosity. Subsequently, the solubility and stability of HMF within these systems were thoroughly investigated.

The set of DESs was chosen on the basis of simple practical considerations. The 15 chosen DESs for the present study derive from the mixture of each HBA with each HBD, are among the most commonly used, are easily available, and are not particularly expensive. ChCl and Bet are benchmark HBAs, just like the three selected HBDs. Pro is also quite popular, while ChAc and Carn are probably less well-known but provide a good comparison point for ChCl and Bet, respectively. This is expected to make the results of the work accessible with a broad range of applicable and general interest. All the mixtures were prepared using a 1:3 ratio between the HBA and the HBD. This choice, although apparently arbitrary, stems from the necessity of a uniform molecular ratio along the whole set of DESs, as reference state, and to avoid the addition of a further degree of variability when interpreting the results. In fact, at the most commonly used 1:2 molar ratio, some of the DESs studied are solid at room temperature. From here on, the concise notation for the DES object of the present study will be used, implying a 1:3 molar ratio.

Finally, it is worth noting that some components—acetate, LA, Car, Bet, and Pro—contain a carboxylic acid functional group that is expected to undergo deprotonation/protonation equilibria depending on the nature of the eutectic mixture partner. Proline can reasonably be assumed to exist predominantly in its zwitterionic form given the p*K*_a__1_ value of 1.95 related to the formation of the carboxylate group, whereas the other components may, in principle, participate in proton transfer processes. FTIR analysis of DES Pro-Gly showed no bands attributable to carboxylic acids, confirming this assumption (spectrum shown in Figure S45). In the context of DES used as solvents for HMF reactions, such equilibria could potentially influence the final outcomes.

Table S1 presents the experimental ^13^C NMR chemical shift values assigned to the carbonyl/carboxyl/carboxylate signals for the entire set of DES (all the spectra are reported in Figures S33–S44). An increase in chemical shift (deshielding) is expected upon deprotonation, whereas a decrease in chemical shift (shielding) can be associated with carboxylate protonation.^35^ Analysis of the experimental ^13^C NMR chemical shifts in Table S1 reveals only minor variations, suggesting that acid–base equilibria play a limited role in these mixtures.

### DES Properties: Reference Systems

To study the solubility and stability of 5-hydroxymethylfurfural in the selected DESs, it was mandatory to first elucidate their physicochemical properties and chemical stability.

Water content is a critical factor when choosing a DES as it can significantly influence its physicochemical properties. Even slight variations in the water content can affect the density and viscosity of DESs, altering their behavior in the desired applications. Moreover, the hygroscopic nature of DESs may lead to a substantial increase in the initial water content according to the storage and handling conditions. Considering an application of the investigated DESs as solvents for HMF under real-world conditions, investigating their water content and water uptake was deemed essential for optimizing their performance in this study.

The water content of the reference system was measured immediately after preparation (*t*_0_) using a standard procedure for all of the mixtures. Most formulations showed values between 0.5 and 0.8 wt % (Table1), except the ChAc-EG one, which can be further dried at higher temperatures if needed (see experimental part). Given that relative humidity is known to influence water sorption in DESs, we measured the water content of all of the DESs after 15 days of exposure to air.^36^ While the ambient relative humidity was not specifically controlled, the water uptake observed reflects the sorption behavior under typical environmental conditions of a synthetic laboratory (see Experimental Section for details).

For all HBAs, the mixture with EG as the HBD showed the highest water uptake, with ChAc-EG at the upper limit (17.1 wt %) and Bet-EG at the lower limit (12.0 wt %). Mixtures with Gly as the HBD showed intermediate water uptake (in the range 6.9–14.5 wt %), while those with LA had lower uptake levels (in the range 4.6–11.6 wt %). Comparing the HBAs, choline chloride and choline acetate resulted in mixtures with the highest water uptake, whereas proline, carnitine, and betaine enabled the formation of less hygroscopic mixtures.

The chemical stability of the blank systems was checked over a thirty-day period by ^1^H NMR spectroscopy (Figures S1–S15). Despite the time variation in between the water contents, all the systems, except one (Pro-EG), remained stable, showing no extra peaks or spectral rearrangements in the NMR profile. Notably, the formulation between proline and ethylene glycol did not result in DES formation: instead, proline recrystallized in the vial upon returning to room temperature with an overall proline loss of 20% with respect to the theoretical calculation (Figure S15).

As mentioned in the Introduction, the preparation of each DES accounts for the same atom economy and E-factor, thus calling for different metrics to address a possible sustainability ranking of the mixtures. Recently, it was shown that the use of the EcoScale provides a first descriptor for sustainability-oriented choice of eutectic mixtures.^37^ The EcoScale approach is based on a simple, semiquantitative evaluation of factors concurring with the environmental profile of a product or a process, assigning penalty points to selected categories such as toxicity, energy consumption, total cost, etc. The penalty points are then subtracted from the reference value of 100, thus giving a final numerical score to be interpreted as “the higher, the greener”. From this viewpoint, the EcoScale parametrization has been defined as “a semi-quantitative tool to select an organic preparation based on both economic and ecological parameters”.^38^ To establish which DES might be more sustainable for potential applications in organic synthesis and to, therefore, discriminate among them, the estimation of the EcoScale parameter was performed in accordance with the literature established procedure (see Tables 1, S2, and S3). All the mixtures present an excellent (>75) score, with values that are similar to each other. The formulations based on carnitine (Carn-Gly, Carn-LA, Carn-EG, Table 1) gave the lowest score due to its relatively high cost compared with the other HBAs considered in the study.

The viscosity (η) and density (ρ) values measured for the investigated eutectic systems are reported in Table 2. As expected, the systems containing glycerol had the highest viscosity. Notably, the formulations containing choline chloride or acetate (ChCl-Gly and ChAc-Gly) present lower viscosity than pure glycerol at 25 °C (η = 970.6 mPa·s)^39^ while all others show an increased value up to 534% when in combination with carnitine. Overall, some indications of intermolecular interactions can be inferred from the data in Table 2. The first is a marked influence of the COO functional group present in the HBA (Bet, Carn and Pro, Figure 1) on the viscosity of the systems containing Gly as the HBD, suggesting a possible role of multicentric H-bonds involving the carboxylate of HBA and hydroxyl groups of the triol. Consistently, the viscosity values of the DES based on the choline cation and glycerol (ChCl-Gly and ChAc-Gly) are lower than those discussed above. A rationale can be found in the composition of the systems: Bet-Gly, Carn-Gly, and Pro-Gly, belonging to the type V class of DES, namely two molecular components (in our case, a zwitterion and a neutral molecular species) directly interacting.

**Table 2:** Viscosity^a^ and Density of Selected DES at 25 °C^b^

substance	viscosity/η (mPa·s)	density/ρ (g/cm^3^)
Bet-Gly^c^	1575.3	1.20430
Bet-LA^c^	515.45	1.15450
Bet-EG^c^	69.84	1.13190
ChCl-Gly^c^	392.68	1.22709
ChCl-LA^c^	156.27	1.13917
ChCl-EG^c^	30.99	1.11439
ChAc-Gly	593.6	1.18514
ChAc-LA	111.11	1.12500
ChAc-EG	49.209	1.10193
Carn-Gly	5190.1	1.23606
Carn-LA	1013.65	1.17209
Carn-EG	431.79	1.16377
Pro-Gly	3795	1.26746
Pro-LA	373.67	1.18394
Gly	970.6^d^	1.2584^e^
LA	solid	1.1335^f^
EG	16.63^g^	1.1091^g^

a A reference guide to viscosity of DES can be found in ref (40).

b The viscosity and density measurement were conducted in triplicated (Err% < 0.08).

c Viscosity and density values from ref (41).

d Viscosity from ref (29).

e Density from ref (42).

f Density from ref (43).

g Viscosity and density from ref (44).

ChCl-Gly and ChAc-Gly are formed by a cholinium salt and a second molecular component. They can be classified as type III DES. In this latter DES-type, the coordination shell arises from the interactions between the HBD and the cation and anion of the HBA, softening the ion pairs leading to a softer network of molecular interactions and a decrease in viscosity. Conversely, the zwitterion nature of Bet, Carn, and Pro HBAs does not allow for cation and anion to move apart, no matter the interactions with the HBD, preventing the viscosity reduction.

The second indication concerns the effect of HBD on the viscosity. This can be inferred by comparing the values of the triads of DES with the same HBA and different HBD, e.g., the systems of type ChCl-Gly, ChCl-LA, ChCl-EG, and the homologous with the other HBAs. The data indicate that the effect on the viscosity follows the order Gly > LA > EG, irrespective of the HBA.

### HMF Stability and Solubility in DES

For all of the different DESs, the solubility and chemical stability of HMF were estimated (Table 3). As for the latter, this was checked over time by ^1^H NMR spectroscopy, to understand if these systems can favor unwanted side-reactions or degradation of the substrate (Figures S16–S31).

**Table 3:** Solubility and Stability of HMF in Each DES Formulation

DES	dissolved HMF (wt %)	*x*_HMF_	HMF stability
Bet-Gly	41	0.24	S
Bet-LA	50	0.48	S
Bet-EG	50	0.38	S
ChCl-Gly	20	0.17	NS
ChCl-LA	43	0.49	S
ChCl-EG	50	0.39	NS
ChAc-Gly	22	0.15	S
ChAc-LA	50	0.50	S
ChAc-EG	50	0.40	S
Carn-Gly	17	0.15	S
Carn-LA	50	0.49	S
Carn-EG	50	0.40	S
Pro-Gly	15	0.12	NS
Pro-LA	33	0.31	NS

In this study, a 50 wt % maximum threshold was set for the dissolution of hydroxymethyl furfural in the solvents. The need to set such a limit is due to the intrinsic instability of HMF itself: a high concentration of HMF in solution might lead to unwanted side reactions such as oligomerization or polymerization. Furthermore, a system was classified as stable if the proton NMR did not highlight the presence of new peaks over time; conversely, it was classified as unstable if any new peaks associated with HMF reactivity were detected.

It can be noticed at first glance that the HBD plays a major role in facilitating the solubility of HMF in the system. The solubilization capability follows the order EG ≥ LA ≫ Gly, regardless of the chemical structure of the associated HBA. Notably, among the chosen hydrogen bond donors, glycerol, despite having three hydrogen bond donor sites that could potentially interact with the carbonyl group of HMF, resulted in the lowest quantity of dissolved HMF usually ranging between 15 and 41 wt % (Bet-Gly, ChCl-Gly, ChAc-Gly, Carn-Gly and Pro-Gly, Table 3), thus confirming the strong tendency of establishing HBA-HBD network, not easily disrupted by HMF.

When considering the influence of the HBA in the solubilization process of HMF, betaine (Bet-Gly, Bet-LA, Bet-EG) proved to be the HBA component that, in combination with any given HBD, dissolved the greatest amount of HMF without causing product degradation or undesired side reactions. These characteristics position betaine as the best candidate for achieving high HMF solubility while also assuring its stability. When considering choline (ChCl-Gly, ChCl-LA, ChCl-EG vs ChAc-Gly, ChAc-LA, ChAc-EG), the acetate counterion seems to favor higher HMF solubility compared to the chloride, with a percentage of dissolved HMF in LA reaching 50% for the acetate and 43% for the chloride (ChCl-LA vs ChAc-LA). This difference might be attributed to the carboxylic group of the acetate, which is more inclined to form hydrogen bond interactions with the hydroxylic group of the solute but also to the higher water content associated with choline acetate. Despite the higher water content of choline acetate containing DES, which might result in the hydration of the aldehydic functional group of HMF, the latter did not show signs of chemical degradation. DES based on carnitine (Carn-Gly, Carn-LA, and Carn-EG) gave results in line with the other hydrogen bond acceptor, while those containing proline (Pro-Gly, Pro-LA) gave the lowest solubility.

**Figure 2:**
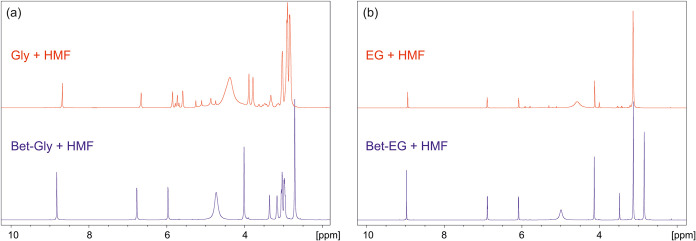
(a) ^1^H NMR comparison of a freshly prepared solution of HMF in glycerol in the presence and absence of betaine, *x*_HMF_ = 0.24; (b) ^1^H NMR comparison of a freshly prepared solution of HMF in ethylene glycol in the presence and absence of betaine, *x*_HMF_ = 0.38.

Furthermore, solubility and stability tests were conducted in pristine glycerol and ethylene glycol. As anticipated, both solvents demonstrated excellent solubility for 5-hydroxymethylfurfural (HMF). However, in the absence of an appropriate hydrogen bond acceptor (HBA), both solvents readily reacted with HMF at room temperature, highlighting its instability under such conditions (Figure 2). Beyond the practical observation that binary mixtures of HBA-EG and HBA-Gly can act as protective agents for HMF, enhancing its chemical stability, we emphasize that the use of such mixtures—regardless of the extent of melting point depression or their classification as “DES”—is expected to significantly influence the solvation properties of HMF.^45^ This, in turn, may lead to the emergence of distinct solvent effects during HMF reactions, potentially altering the reaction pathways and selectivities.

**Scheme 1:**
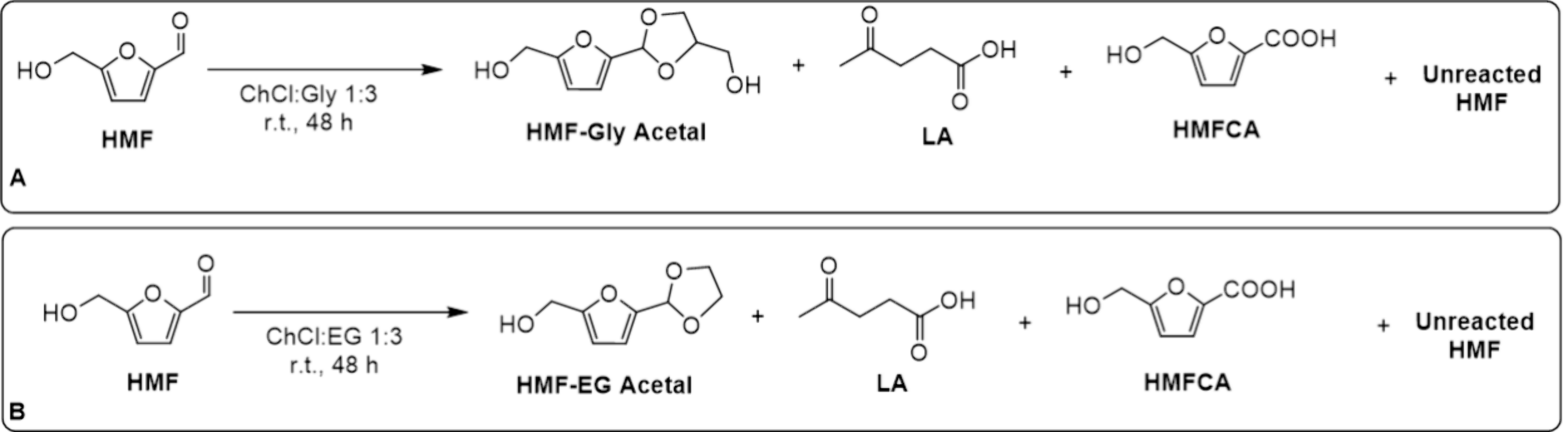
HMF-DES Side Reaction Products (a) in the Presence of Choline Chloride and Glycerol, (b) in the Presence of Choline Chloride and Ethylene Glycol.

Noticeably, many of the formulations provided a chemical environment in which HMF remained stable for up to one month (Table 3), according to the NMR analyses. Choline chloride in combination with both glycerol and ethylene glycol (ChCl-Gly and ChCl-LA) enhances HMF reactivity, quickly converting part of it into the corresponding degradation, oxidation, and condensation products within 24–72 h (Scheme 1, Figures S22, and S25).

A brief description of the byproduct identification is now given. These were identified by ESI–MS and ^1^H NMR of the corresponding HMF-DES mixture after extraction with ethyl acetate (Figures S23 and S26). To gain a clearer understanding of the role of HBAs and HBDs in the dissolution of HMF, two blank tests were also conducted using glycerol and ethylene glycol. As anticipated, both acted as solvents capable of dissolving HMF even without the HBA. However, as already discussed, they also showed a tendency to readily react with HMF (Scheme 1).

Table 4 summarizes the spectral peaks, relative abundance, and tentative peak assignment in the case of ChCl-Gly and ChCl-EG (Table 1, Scheme 1). Blank systems HMF-Gly and HMF-EG are also reported for comparison, along with the reference fragments for a methanolic solution of pure HMF.

**Table 4:** Summary of ESI–MS Data for HMF, HMF Solutions in ChCl-Gly, ChCl-EG, and for the Corresponding HMF Solutions in the Systems without the HBA Choline Chloride (Gly-HMF and EG-HMF)

positive ion polarity						
*m*/*z*	Rel abundance (%) HMF^a^	Rel abundance (%) in GLY+HMF^b^	Rel abundance (%) in ChCl-Gly+HMF^c^	Rel abundance (%) in EG+HMF^b^	Rel abundance (%) in ChCl-EG+HMF^c^	assignment^d^
104			100		100	Ch^+^
149	100	5	7	100	trace	[HMF + Na]^+^
181	12	trace	trace	13	trace	[HMF + MeOH + Na]^+^
183		3	4			[LA + EG – H_2_O + Na]^+^
193				11	8	[HMF-EG Acetal + Na]^+^
209				5		[HMFCA + EG – H_2_O + Na]^+^
223		100	36			[HMF-Gly Acetal + Na]^+^
239		10	11			[HMFCA + Gly – H_2_O + Na]^+^
255				13	3	[HMF + 2(EG) – H_2_O + Na]^+^
257^e^	10					[2(HMF) – H_2_O + Na]^+^
257^f^			13			[HMFCA + Gly + Na]^+^or [2(HMF) – H_2_O + Na]^+^
271				5		[HMF + 2(EG) – H_2_O + K]^+^
273			6			[HMFCA + Gly + K]^+^
289			4			[2(HMFCA) – H_2_O + Na]^+^
315		5				[HMF + 2(Gly) – H_2_O + Na]^+^
331			21			[HMFCA + 2(Gly) – H_2_O + Na]^+^
347			10			[HMFCA + 2(Gly) – H_2_O + K]^+^

negative ion polarity						
*m*/*z*	Rel abundance (%) HMF^a^	Rel abundance (%) in GLY+HMF	Rel abundance (%) in ChCl-Gly+HMF^b^	Rel abundance (%) in EG+HMF	Rel abundance (%) in ChCl-EG+HMF^b^	assignment^d^
115		10	95	4	41	[LA – H]^−^
125	100					[HMF – H]^−^
141		4	11	11	92	[HMFCA – H]^−^
157	39					[HMF + MeOH – H]^−^
159					16	[LA + EG – H_2_O – H]^−^
174^g^					100	[ChCl + Cl]^−^
189		21	100			[LA + Gly – H_2_O – H]^−^
283		58	8	52	25	[2(HMFCA) – H]^−^

a This column refers to ESI–MS data of a methanolic solution of pure HMF.

b These two columns refer to the ESI–MS spectra carried out on the solution of HMF with the HBD component only (glycerol and ethylene glycol).

c These columns describe the ESI–MS spectra of HMF in the presence of both HBD and HBA.

d This column shows the peak assignment. The abbreviations used are introduced in Figure 1 and Scheme 1. Sodium and potassium ions are environmental.

e The structure of this ion matched the DP-1 of ref (46). As the HMF spectrum in the negative ion polarity did not show peaks consistent to HMFCA species, the peak at *m*/*z* 257 of the positive polarity mode is assigned to the Na cationized and dehydrated HMF dimer only.

f In this case, the negative ion polarity mode shows the presence of HMFCA in the spectrum of HMF in ChCl-Gly. Thus, the *m*/*z* 257 in the positive ion mode is consistent with both the assignments proposed.

g Isotopic cluster consistent with 2 Cl in the elemental formula.

The solution of HMF in ChCl-Gly shows the peaks at *m*/*z* 104 (rel. ab 100%, base peak) corresponding to cholinium ion (Ch^+^), *m*/*z* 149 (7), corresponding to Na^+^ cationized HMF, *m*/*z* 223 (36), assigned to the species [HMF-Gly Acetal + Na]^+^, the Na^+^ cationized form of the acetal of Scheme 1A. The peak at *m*/*z* 223 is the base peak in the absence of ChCl (see the second column of Table 4), and it provides experimental evidence of the reaction of HMF and glycerol as one of the possible pathways accounting for HMF instability. Interestingly, the acetal of HMF with EG (Scheme 1B, abbreviated as HMF-EG Acetal) was also observed at *m*/*z* 193 in the ESI spectra of HMF dissolved in EG and in ChCl-EG, although with a lower relative abundance compared to the Gly homologues. A second byproduct of HMF was identified by assigning the peak at *m*/*z* 239 to the noncovalent association of 5-hydroxymethylfuranoic acid (HMFCA, see Scheme 1) with glycerol, followed by the in-source elimination of one neutral H_2_O molecule and Na^+^ attachment. The corresponding adduct in the case of EG was observed at *m*/*z* 209 only in the absence of ChCl and with a low relative abundance. The existence of HMFCA was double-checked by recording the negative ions polarity ESI MS spectrum, where the signals at *m*/*z* 141 (11) can be confidently assigned to the conjugate base of HMFCA. The formation of HMFCA was detected in all of the solutions, with and without ChCl, with a high relative abundance in the case of the solution of HMF in ChCl-EG. The detection of an oxidation byproduct of HMF is novel and quite unexpected. Recent data published by Lu et al.^46^ describe the degradation of HMF in aqueous solution under various chemical stress conditions, including hydrolysis at neutral, acidic, and alkaline pH, oxidation, thermal decomposition, and UV- and visible-light irradiation. However, the authors did not report any decomposition product corresponding to HMF derivatives featuring a carboxylic group derived from the formyl moiety. The discrepancy between Lu et al.’s findings and ours may be attributed to the different solvents used—aqueous solution versus DES—highlighting an intriguing aspect that warrants further investigation. Additionally, the decomposition product corresponding to *m*/*z* 257 in Table 4 aligns perfectly with the decomposition species DP-1 reported by Lu et al.

The most intense peaks in the negative polarity ion ESI MS of the solution HMF in ChCl-Gly are observed at *m*/*z* 115 (95) and *m*/*z* 189 (100). These two signals are consistent with the conjugate base of LA and the adduct of dissociated LA with dehydrated glycerol. Levulinic acid LA is known to be one of the main decomposition products of HMF,^47−49^ along with formic acid, which, however, was not detected in the ESI experiments. The control experiment carried out on the solution containing HMF and Gly only gave the same two peaks with a much lower relative abundance. A similar pattern for *m*/*z* 115 was observed in EG, although with lower relative abundance with respect to Gly, while the peak at *m*/*z* 159, formally attributed to [LA + EG – H_2_O – H]^−^, was observed only in the HMF solution in ChCl-EG.

All in all, the ESI–MS results allowed us to identify LA, HMFCA, and the HMF acetals as the main decomposition products in the presence of Gly and EG. While the pathways for acetal formation and the degradation of HMF to LA are well established or already documented in the literature,^47−49^ the origin of the unprecedented oxidation products identified in this study remains unclear and requires further experimental investigation. Therefore, at this stage, we refrain from proposing a mechanistic hypothesis to avoid unwarranted speculation.

Considering that HMF resulted in being stable over time in the systems containing ChAc, the stability data seem to indicate that the nature of the pairing anion plays a pivotal role in promoting the HMF reactions. This is not completely unexpected considering the effect of the anion on the pH of choline-containing DESs and the sensitivity of HMF to pH variations.

In the context of HMF instability in the presence of proline (Pro-Gly and Pro-LA, Table 3), ^1^H NMR analyses of the mixtures revealed a notable transition from sharp peaks to broader bands indicative of a chemical transformation. This spectral change was accompanied by a darkening of the solution, shifting from a light orange to a deep brown hue. Literature supports that prolonged storage of HMF leads to degradation, which is often observable through such color changes. The degradation products include HMF condensation products, which range from dimers to oligomers. Additionally, the aging of HMF is associated with the formation of humins, complex polymeric structures that arise from condensation reactions.^19,46,50^

## Conclusions

This study demonstrated the potential of 15 deep eutectic solvents (DESs) for dissolving and, in most cases, stabilizing 5-hydroxymethylfurfural (HMF), offering valuable insights into sustainable media for valorizing biomass-derived platform chemicals. The systems were evaluated using parameters such as water content, water absorption, green EcoScale scores, chemical stability, density, and viscosity. DESs containing levulinic acid and ethylene glycol exhibited the highest HMF solubility, except when proline was used as the hydrogen bond acceptor (HBA), which drastically reduced the solubility.

HMF stability was generally well-maintained across most DESs, with notable exceptions in those containing proline and choline chloride, which promoted side reactions. Chloride ions facilitated HMF oxidation to 5-hydroxymethyl-2-furancarboxylic acid or its degradation to levulinic acid. This study further revealed the dual role of HBAs, which can either stabilize HMF or enhance its reactivity, underscoring the importance of careful HBA selection based on desired outcomes.

This study highlighted the properties of several binary systems based on liquid hydrogen bond donors (HBDs), such as glycerol (Gly) and ethylene glycol (EG). While some in the scientific community remain skeptical about distinguishing these systems from conventional solutions of hydrogen bond acceptors (HBAs) (e.g., ChCl) in diols or triols (such as EG or Gly), the ability of choline derivatives to modulate the solvation properties of EG has been elegantly demonstrated by Klein et al.^51^

The emerging perspective is that binary (eutectic) mixtures function as genuine “designer solvents,” where both components act synergistically to shape the solvation environment of the substrate. In the context of this study, such tailored solvation properties can have a significant impact. Specifically, the complex and tunable solvation mechanisms of these systems can influence the transition state of reactions occurring in DES, allowing chemists to guide competitive reactions under kinetic control. This, in turn, enables selective product formation by preferential stabilization of specific transition states through targeted solvation effects.

Betaine-based DES formulations have emerged as particularly advantageous, combining higher HMF solubility with enhanced stability. Their green EcoScale scores, slightly below 100, position them as environmentally sustainable options for HMF dissolution, strongly aligning with green chemistry principles.

In summary, this research advances our understanding of HMF-DES systems and underscores their potential for sustainable and efficient biomass-derived chemical processing. Type V DESs, particularly those using betaine as the HBA, exhibit significant promise for stabilizing HMF and unlocking its full potential. These findings position DESs as valuable, eco-friendly tools for advancing green chemical processes involving HMF.

By integrating environmentally benign solvents with renewable feedstocks, this study exemplifies key principles of green chemistry, emphasizing its role in sustainable chemical design. Waste Prevention is achieved through the null E factor and 100% atom economy in DES preparation. The use of Safer Solvents and Auxiliaries is inherent in the formulation employed, while the mild preparation conditions adhere to the Design for Energy Efficiency principle. The combination of HMF, a renewable synthon, and DES as solvents aligns with the Use of Renewable Feedstocks. Additionally, as DESs are inherently biodegradable, their environmental impact is minimized postuse, supporting the principle of Design for Degradation.

## Experimental Section

### Materials and Methods

HMF (98%) was purchased from Fluorochem, l-proline and L-carnitine were purchased from BLD Pharm, and all the other reagents and solvents were purchased from Merck and used without any further purification if not otherwise stated in the manuscript.

^1^H NMR spectra were recorded at 298 K on a Bruker NEO 500 MHz spectrometer equipped with a direct observe BBFO (broadband including fluorine) iProbe and a variable-temperature unit. The instrument was carefully tuned, shimmed, and the 90° pulses calibrated. For all the samples, ^1^H NMR spectra were recorded with 16 scans and using 32k points.

ESI–MS spectra were collected on a Bruker Esquire 3000+ instrument equipped with an electrospray ionization source and quadrupole ion trap detector. The samples were diluted in methanol to 10 2 g/L and directly infused into the spectrometer source at a 4 μL/min rate. The analyses were performed in positive and negative ion mode after optimization of the acquisition parameters: 4.5 kV needle voltage, 10 L/h N_2_ flow rate, 40 V cone voltage, trap drive set to 46, 115.8 V capillary exit, 13,000 (*m*/*z*)/s scan resolution over the 35–900 *m*/*z* mass/charge range.

Water content was measured through a Karl Fisher titration method performed with an MKC-710 B instrument by KEM Kyoto Electronics.

#### General Procedure for Water Content Measurement of the Reference DES Systems

All the reactants were kept under vacuum at 2 × 10^–3^ mbar and at room temperature for 16 h in a dryer, and were kept until used. The desired amount of HBA and HBD was weighed in a vial equipped with a magnetic stirrer, sealed, and heated to 50 °C until a homogeneous liquid system was obtained. All the mixtures appear as colorless liquids except for the one with proline, which has a characteristic light-yellow color. The flask was brought back to room temperature, and the water content was measured right after the preparation. All the prepared blank systems were kept in open vials for 15 days, then the water content was remeasured to evaluate the water uptake of the DESs.

As a reference for “ambient conditions”, suffice here to report the average lab *T* = 19 °C and that the average relative humidity recorded in Milano’s area in the period of measurement was in the 77 to 95% range.

The result reported in Table 1 for each batch is the average number for the triplicate measurement.

The ChAc-EG DES, with a water content >1 wt %, can be further dried to reduce the water content to 0.5 wt % by heating the system at 80 °C for 8–10 h.

#### General Procedure for DES Preparation and HMF Solubility Study

All the HBAs and HBDs were kept under vacuum at 2 × 10^–3^ mbar and room temperature in a dryer for 16 h and were kept until used. The desired amount of HBA and HBD was weighed in a two-neck round-bottom flask equipped with a magnetic stirrer and kept at 50 °C under an inert atmosphere until a homogeneous colorless solution was obtained.

The homogeneous mixture was brought back to room temperature, and then, HMF was added to the solution, under N_2_, until the formation of a saturated solution or until 50 wt % of HMF was achieved in the solvent.

#### General Procedure for DES and DES-HMF Mixture Chemical Stability Studies

All of the prepared solutions (blank solutions and DES-HMF mixtures) were transferred, after preparation, to 5 mm NMR tubes, equipped with a coaxial insert containing deuterated dimethylsulfoxide (DMSO-*d*_6_) and tetramethylsilane (TMS), as a lock signal and chemical shift reference, respectively. This was kept sealed and checked over time for 30 days by ^1^H NMR.

#### Density Measurements

Densities of DESs at different temperatures were measured using a density meter (Anton Paar, DMA 4500 M). This instrument exploits a U-shaped oscillating tube as a sensing element. Measurements were collected at a temperature of 25 °C. Densimeter calibration was conducted by using the reference density values of water, which were obtained from the fundamental equation of state by Wagner and Pruss (uncertainty lower than ± 0.003% in the full pressure and temperature ranges).

#### Viscosity Measurements

Viscosities of DESs as a function of temperature were measured by using a modular compact rheometer (MCR 302, Anton Paar) equipped with a plate–plate geometry (diameter of 5 cm) and a protective hood. Before conducting the measurements, the samples were subjected to a preshear to get uniform and homogeneous samples on the plate. First of all, flow curve measurements were carried out by varying the shear rate from 1 to 1000 s^–1^ at 25 °C and 1 to 100 s^–1^ at 25 °C for carnitine:glycerol. Thirty data points were collected by the rheometer every 10 s. For non-Newtonian liquids, the measurements of viscosity were extrapolated from the flow curve at 50 s^–1^. The temperature of the instrument was controlled by a Water-Cooled Peltier system (H-PTD200, Anton Paar).

## Supporting Information Available

^1^H and ^13^C-{^1^H} NMR spectra for all mixtures, FT-IR spectrum of Pro-Gly, Tables of ^13^C chemical shifts, and EcoScale calculation (PDF)
