# Supplementary material for: Harnessing the Potential of 5-Hydroxymethylfurfural: Investigating Solubility and Stability in Tailored Deep Eutectic Solvents
Source: ACS Sustain Chem Eng. 2025 Apr 2;13(18):6518–27. doi: 10.1021/acssuschemeng.4c10788 (PMC12077263; doi:10.1021/acssuschemeng.4c10788)
Supplement: Supplementary file 1 — sc4c10788_si_001.pdf [file sc4c10788_si_001.pdf]

Supporting Information

Harnessing the Potential of 5-Hydroxymethylfurfural: Investigating Solubility and Stability in Tailored Deep Eutectic Solvents

*Grazia Isa C. Righetti†, Maria Enrica Di Pietro†, Gabriella Leonardi†, Arianna Sinibaldi‡, Andrea Mezzetta‡, Lorenzo Guazzelli‡ and Andrea Mele†**

† *Department of Chemistry, Materials and Chemical Engineering "G. Natta", Politecnico di Milano, Piazza L. da Vinci 32, 20133 Milano, Italy.*

‡ *Università di Pisa, Dipartimento di Farmacia, via Bonanno 6, 56126 Pisa, Italy.*

* Andrea Mele - Email: andrea.mele@polimi.it

Number of pages: 26

Number of figures: 44

Number of tables: 3

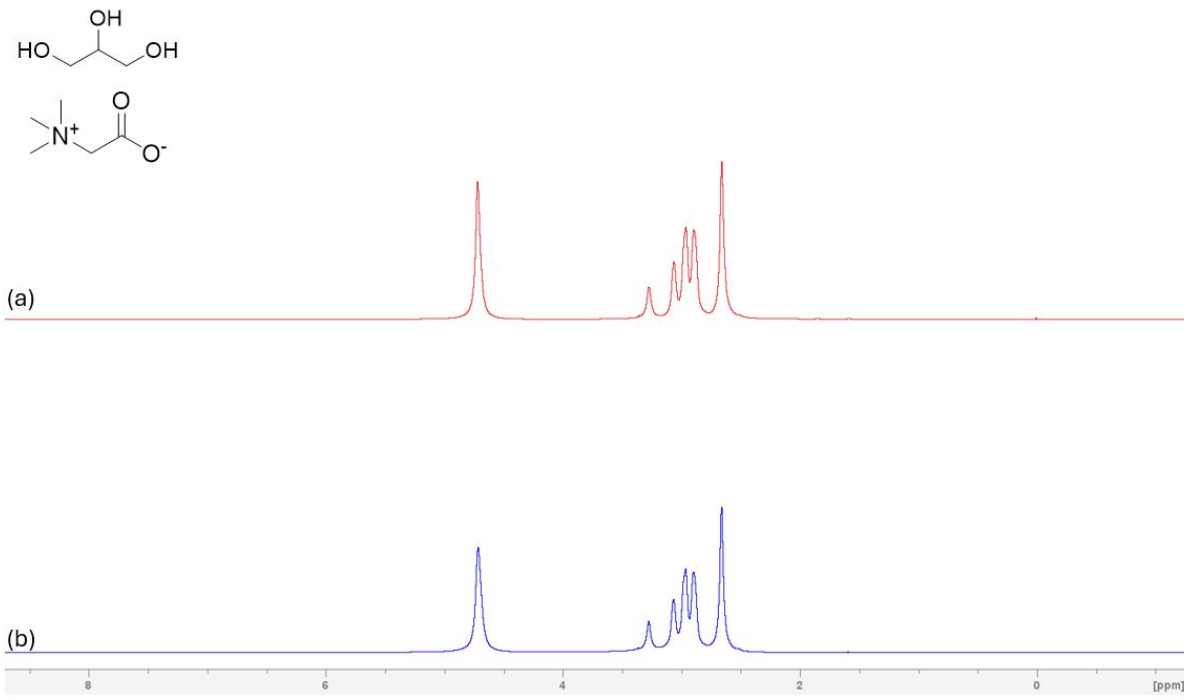

Figure S.1 ^{1}H-NMR in DMSO-d6 (coaxial inset) of Bet-Gly mixture (a) freshly prepared mixture and (b) same mixture after 30 days

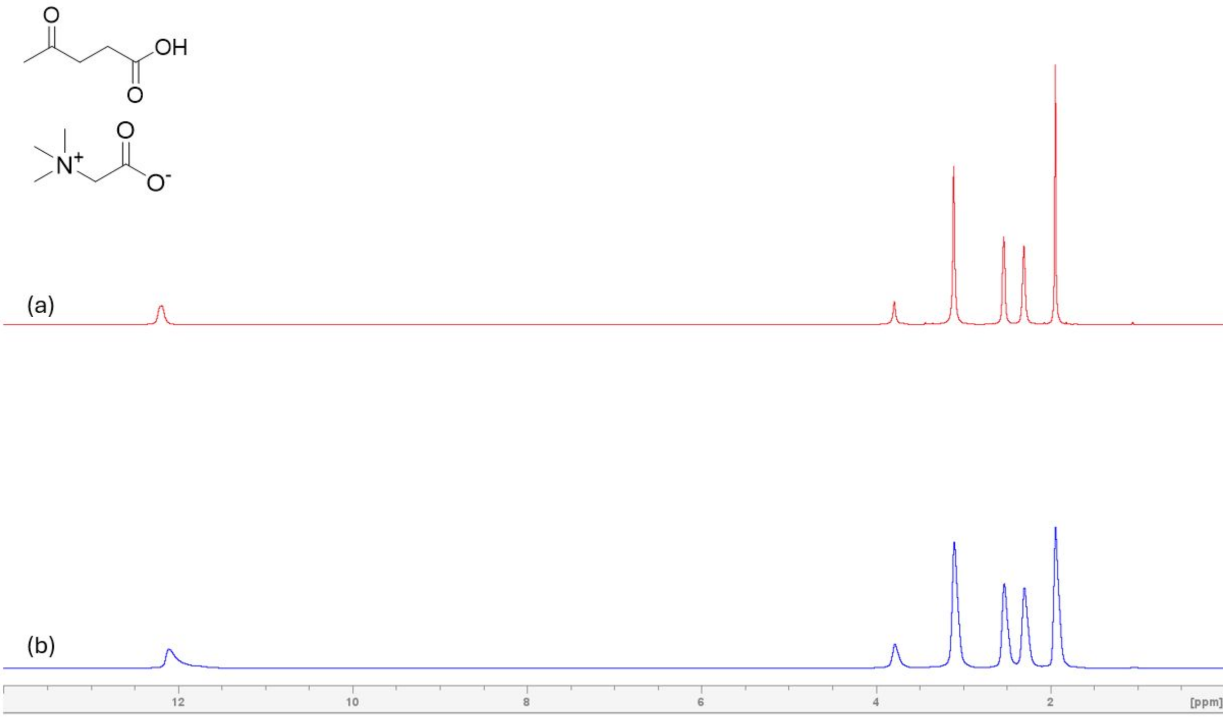

Figure S.2 ^{1}H-NMR in DMSO-d6 (coaxial inset) of Bet-LA mixture (a) freshly prepared mixture and (b) same mixture after 30 days

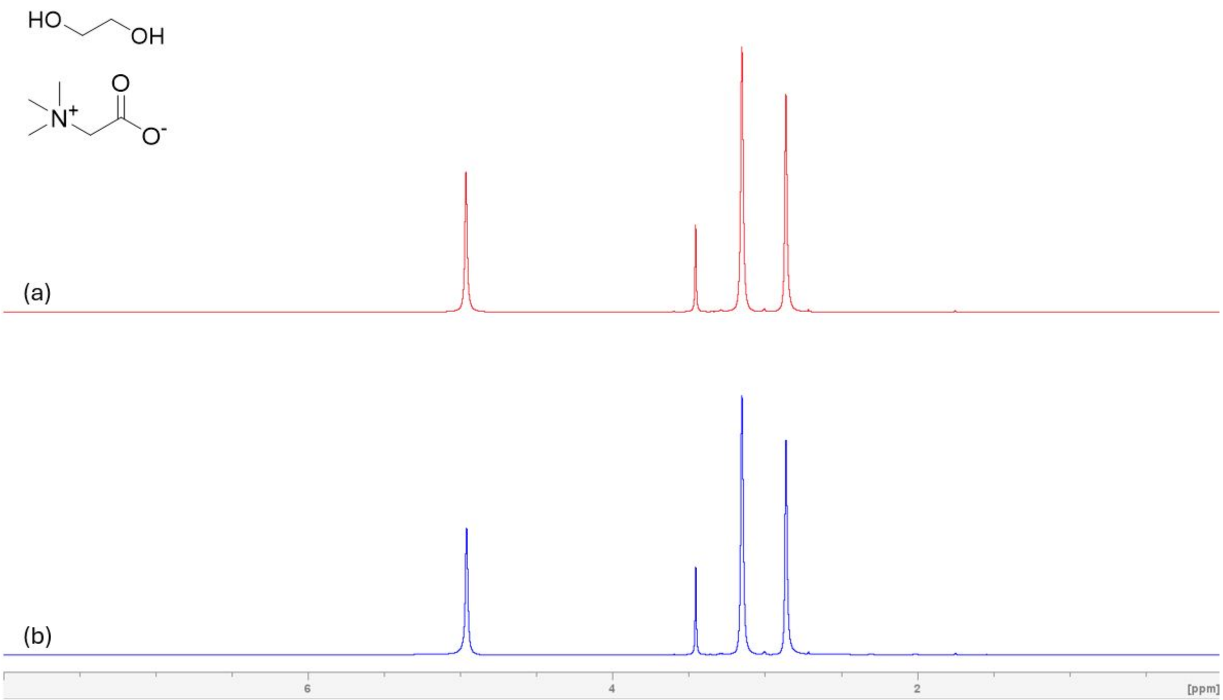

Figure S.3 ^{1}H-NMR in DMSO-d6 (coaxial inset) of Bet-EG mixture (a) freshly prepared mixture and (b) same mixture after 30 days

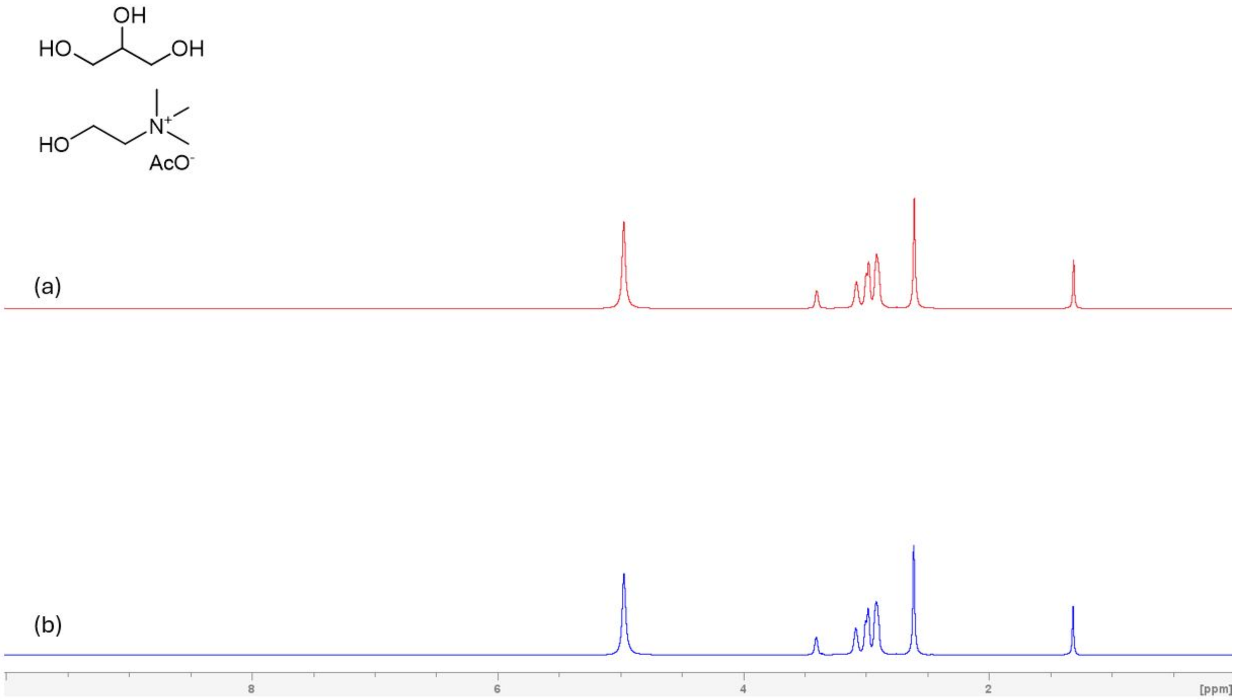

Figure S.4 ^{1}H-NMR in DMSO-d6 (coaxial inset) of ChAc-Gly mixture (a) freshly prepared mixture and (b) same mixture after 30 days

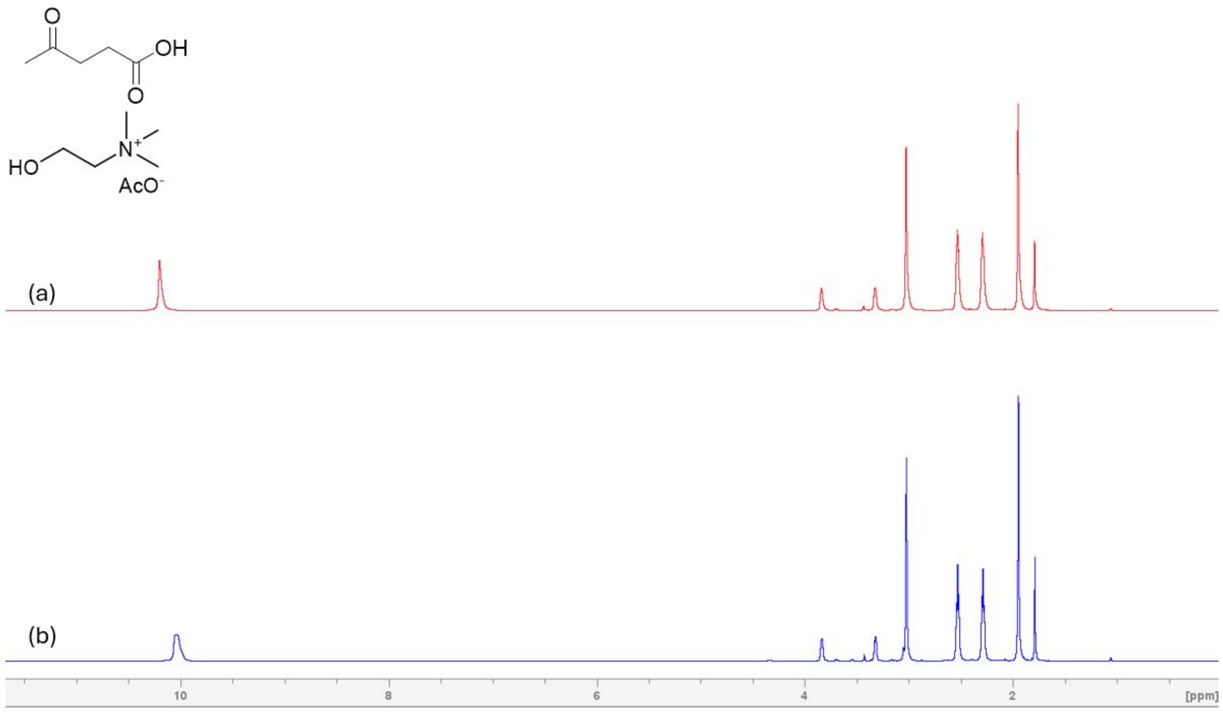

Figure S.5 ^{1}H-NMR in DMSO-d6 (coaxial inset) of ChAc-LA mixture (a) freshly prepared mixture and (b) same mixture after 30 days

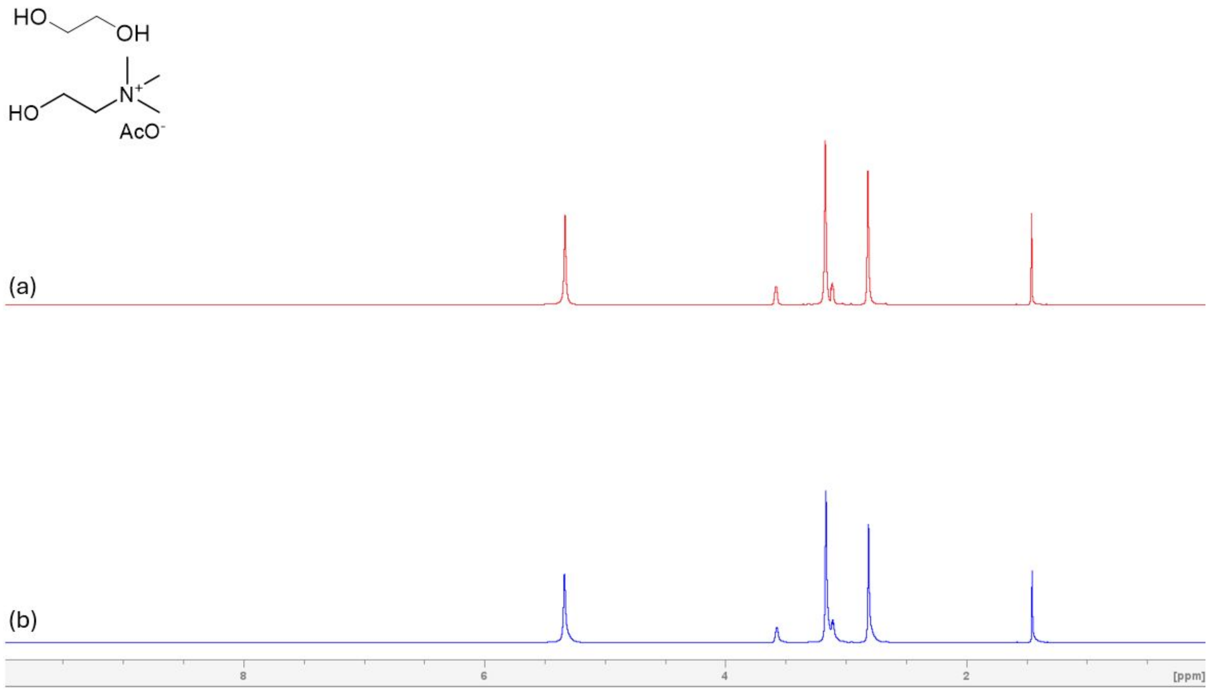

Figure S.6 ^{1}H-NMR in DMSO-d6 (coaxial inset) of ChAc-EG mixture (a) freshly prepared mixture and (b) same mixture after 30 days

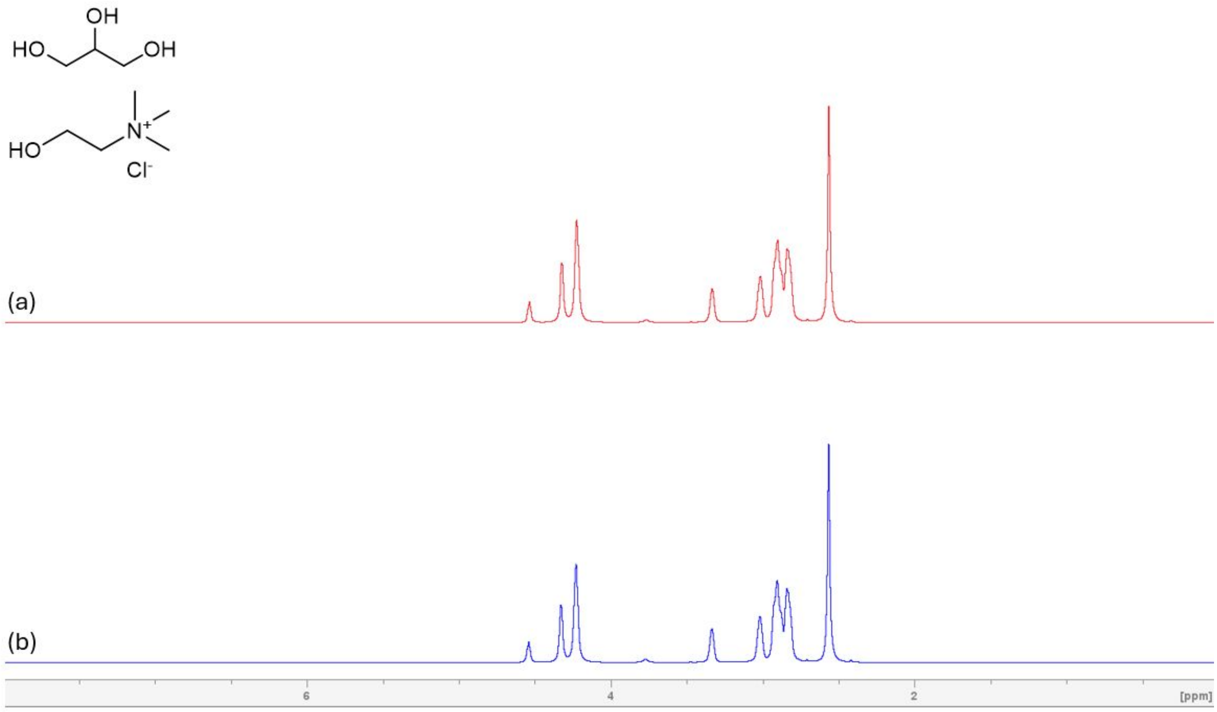

Figure S.7 ^{1}H-NMR in DMSO-d6 (coaxial inset) of ChCl-Gly mixture (a) freshly prepared mixture and (b) same mixture after 30 days

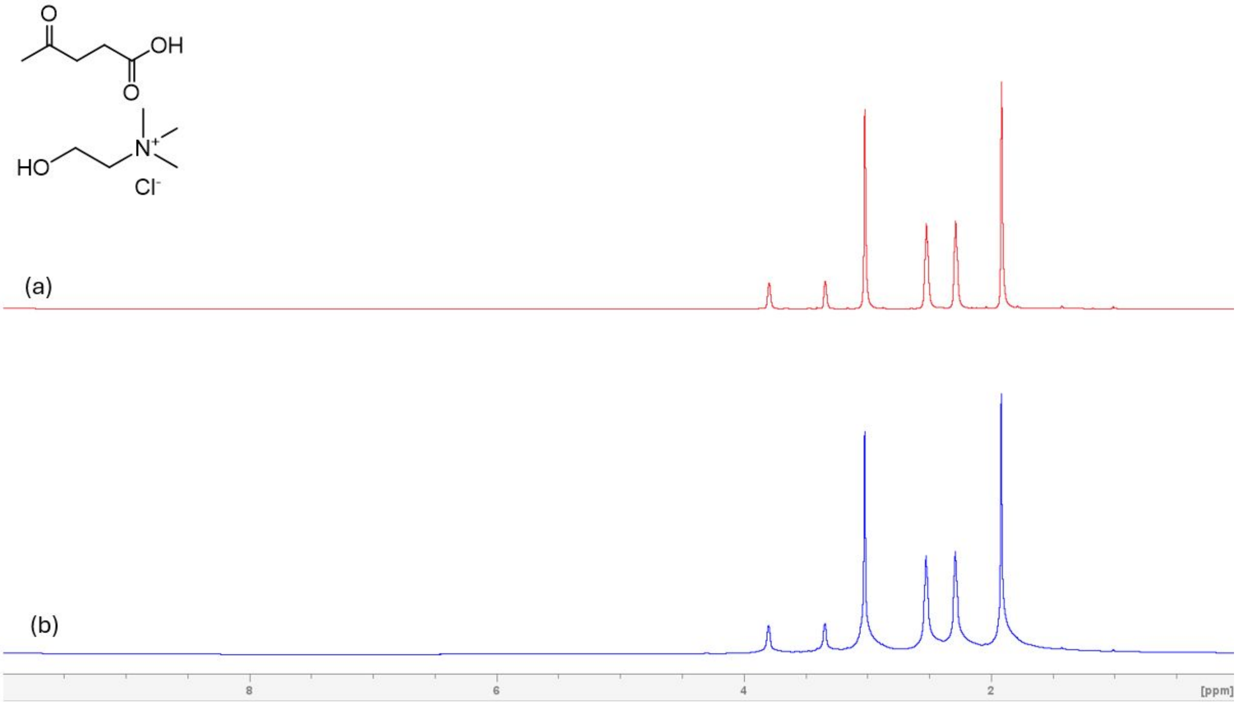

Figure S.8 ^{1}H-NMR in DMSO-d6 (coaxial inset) of ChCl-LA mixture (a) freshly prepared mixture and (b) same mixture after 30 days

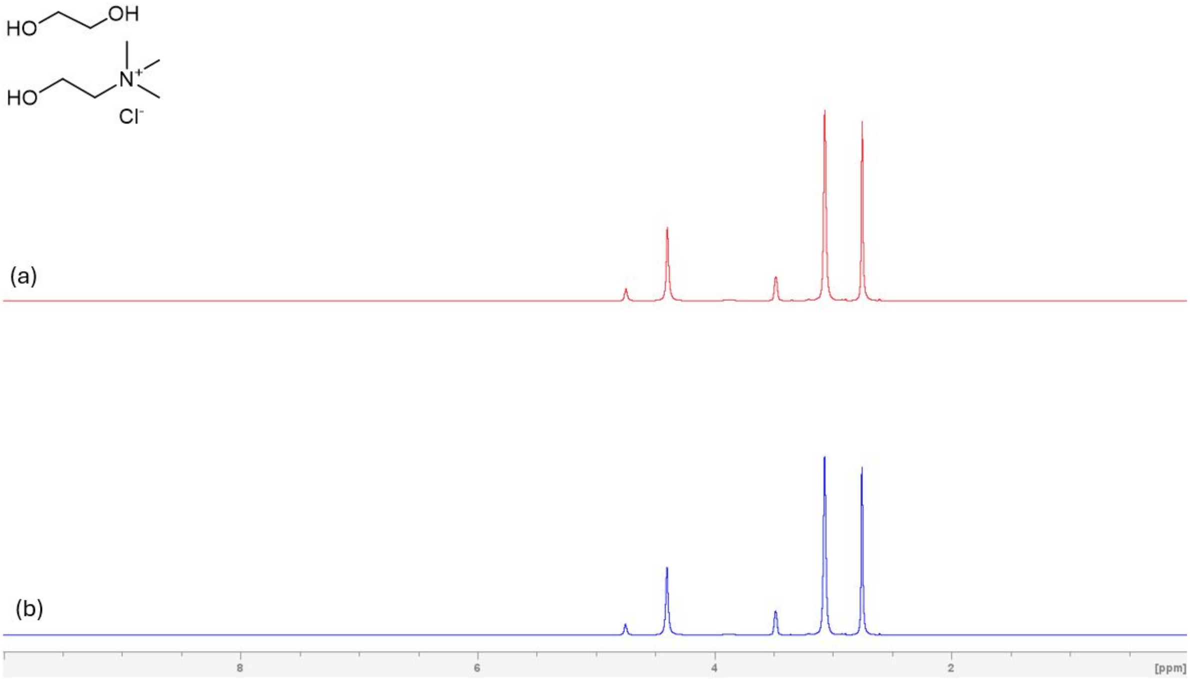

Figure S.9 ^{1}H-NMR in DMSO-d6 (coaxial inset) of ChCl-EG mixture (a) freshly prepared mixture and (b) same mixture after 30 days

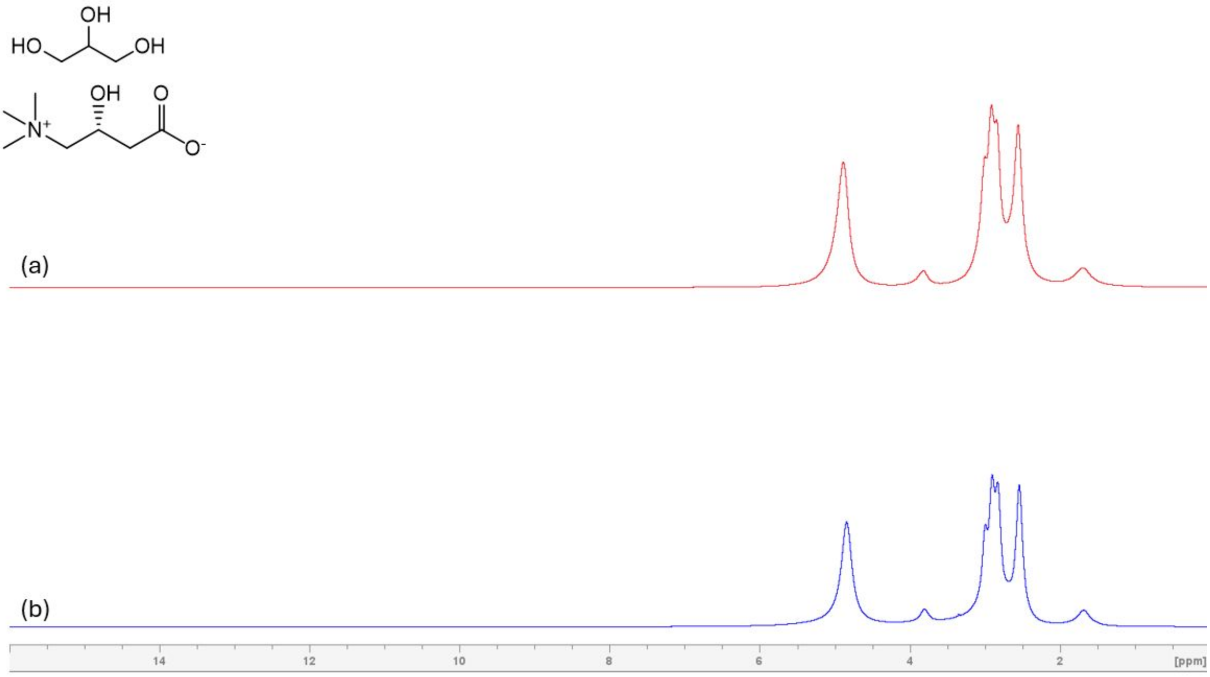

Figure S.10 ^{1}H-NMR in DMSO-d6 (coaxial inset) of Carn-Gly mixture (a) freshly prepared mixture and (b) same mixture after 30 days

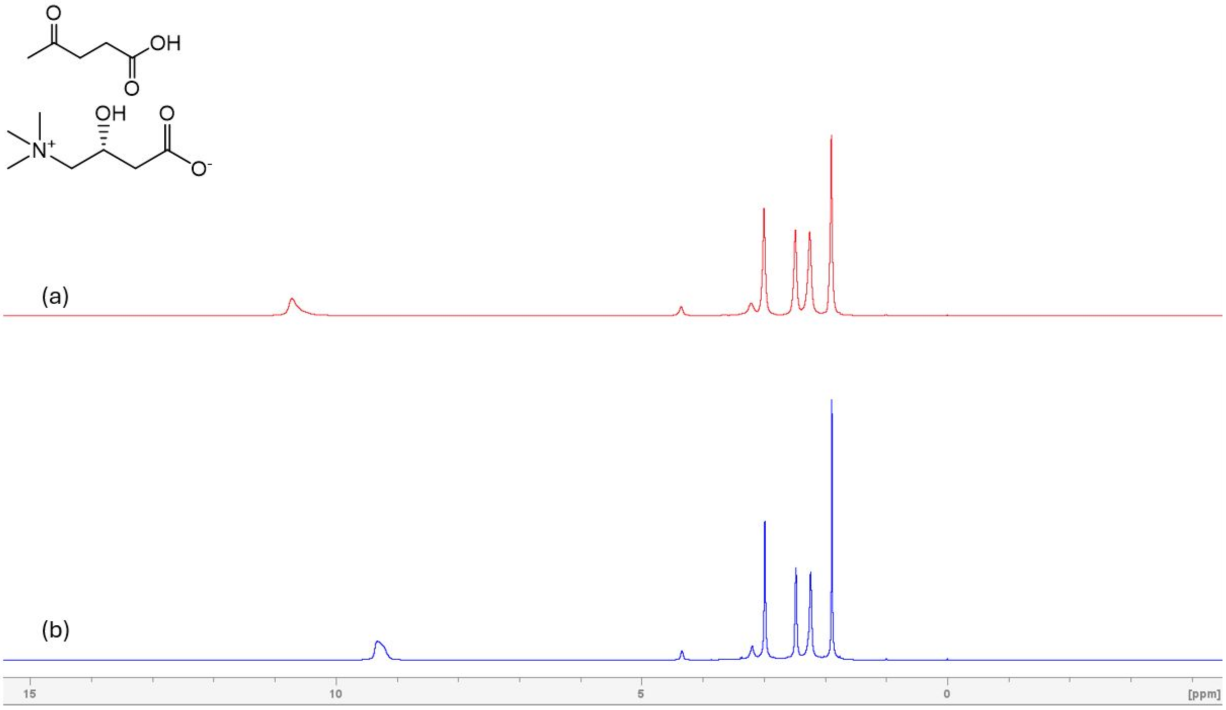

Figure S.11 ^{1}H-NMR in DMSO-d6 (coaxial inset) of Carn-LA mixture (a) freshly prepared mixture and (b) same mixture after 30 days

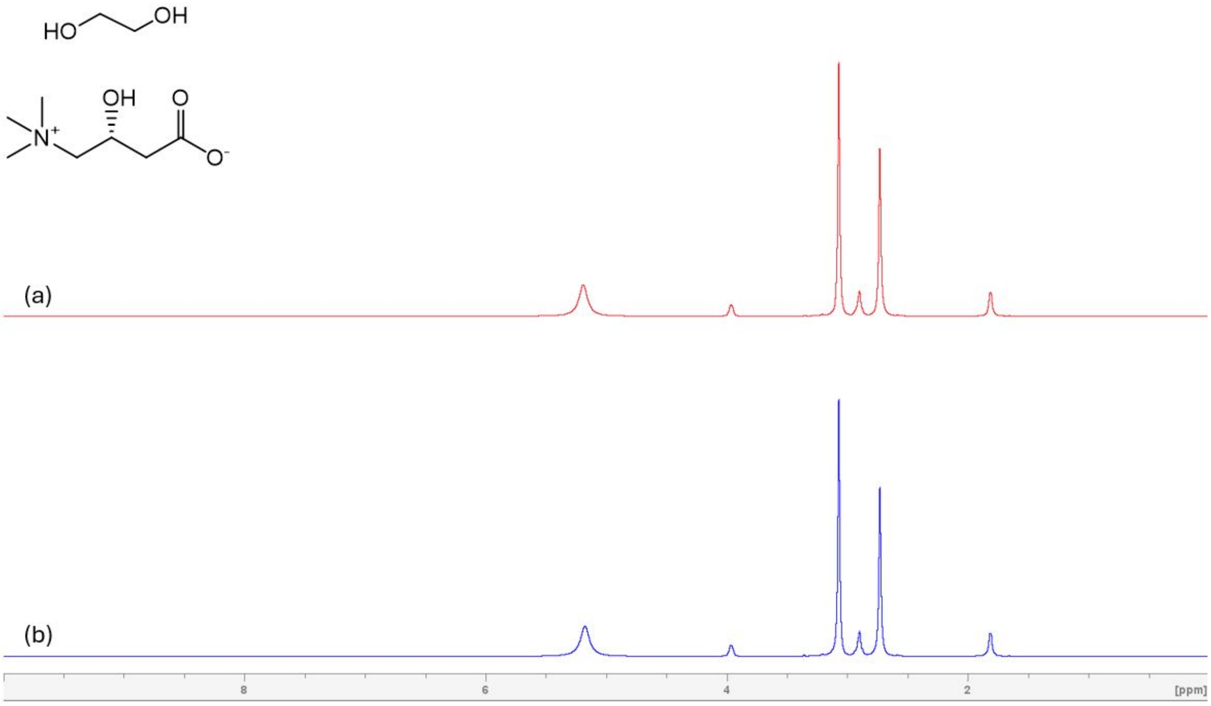

Figure S.12 ^{1}H-NMR in DMSO-d6 (coaxial inset) of Carn-EG mixture (a) freshly prepared mixture and (b) same mixture after 30 days

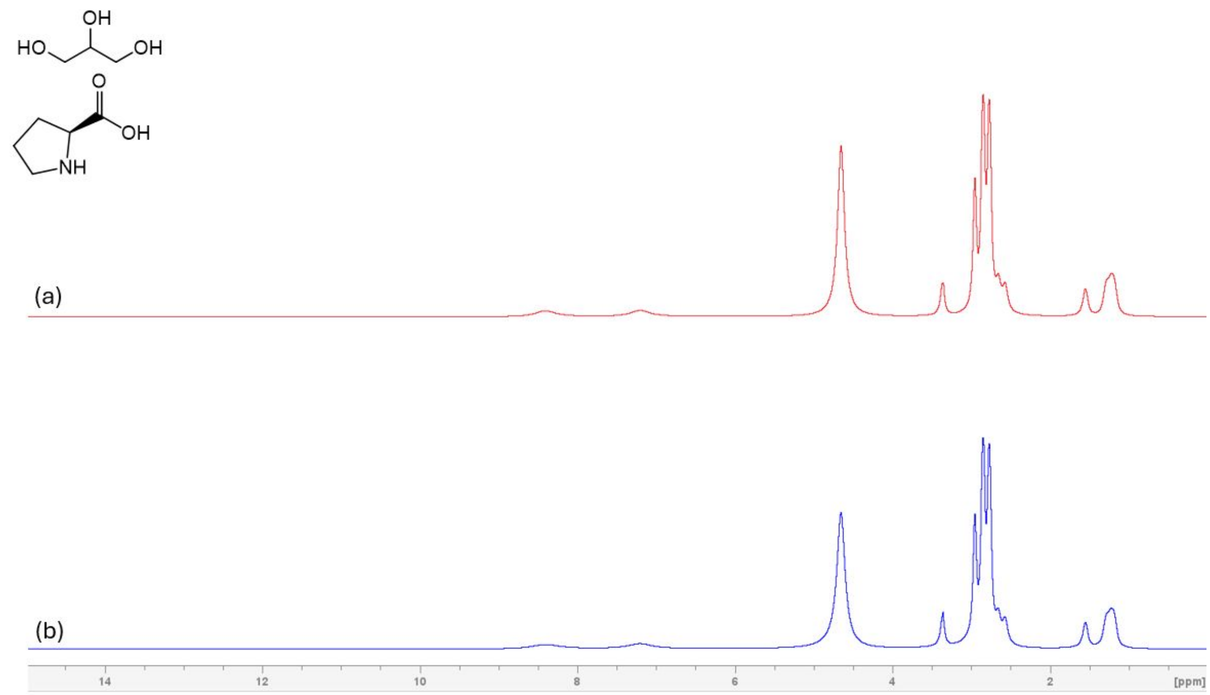

Figure S.13 ^{1}H-NMR in DMSO-d6 (coaxial inset) of Pro-Gly mixture (a) freshly prepared mixture and (b) same mixture after 30 days

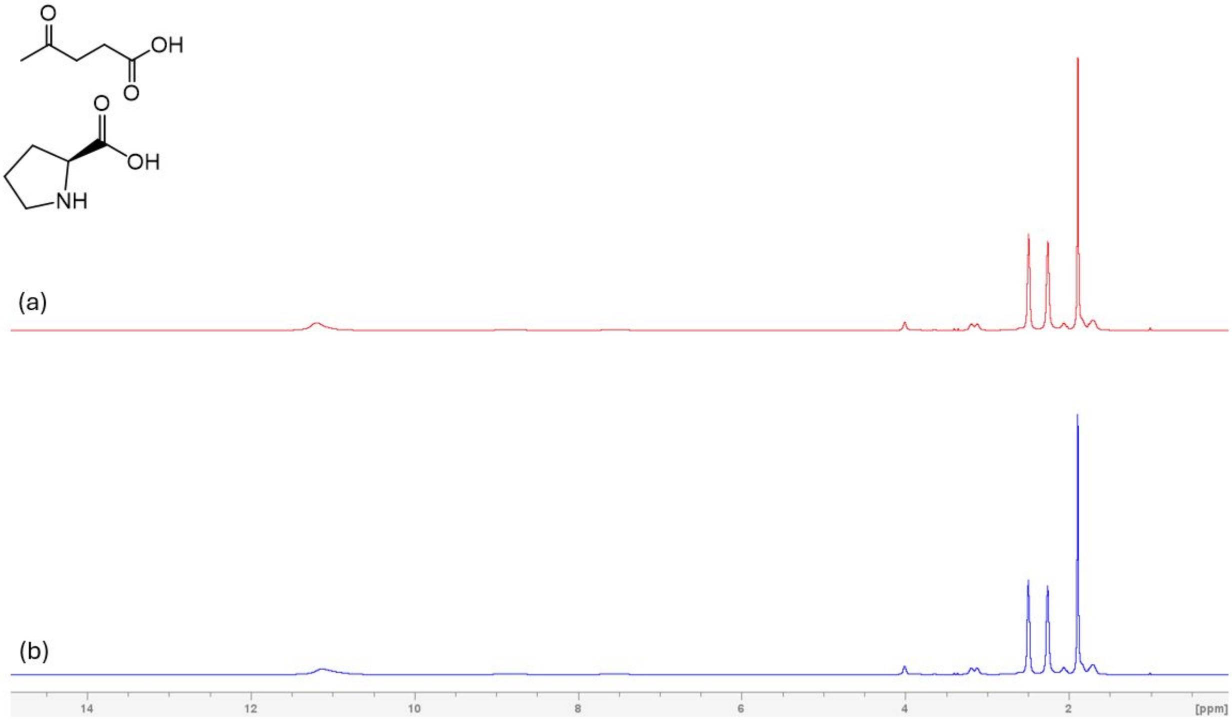

Figure S.14 ^{1}H-NMR in DMSO-d6 (coaxial inset) of Pro-LA mixture (a) freshly prepared mixture and (b) same mixture after 30 days

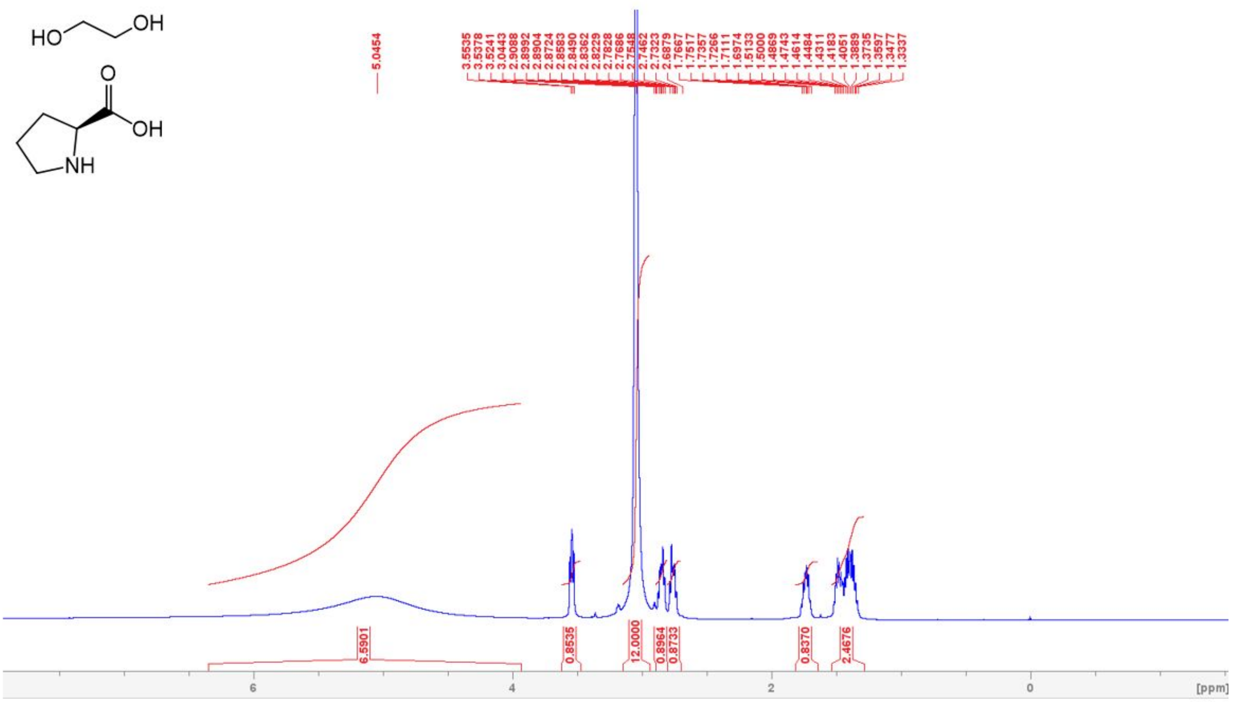

Figure S.15 ^{1}H-NMR in DMSO-d6 (coaxial inset) of Pro-EG mixture (biphasic system with Pro crystalized in NMR tube)

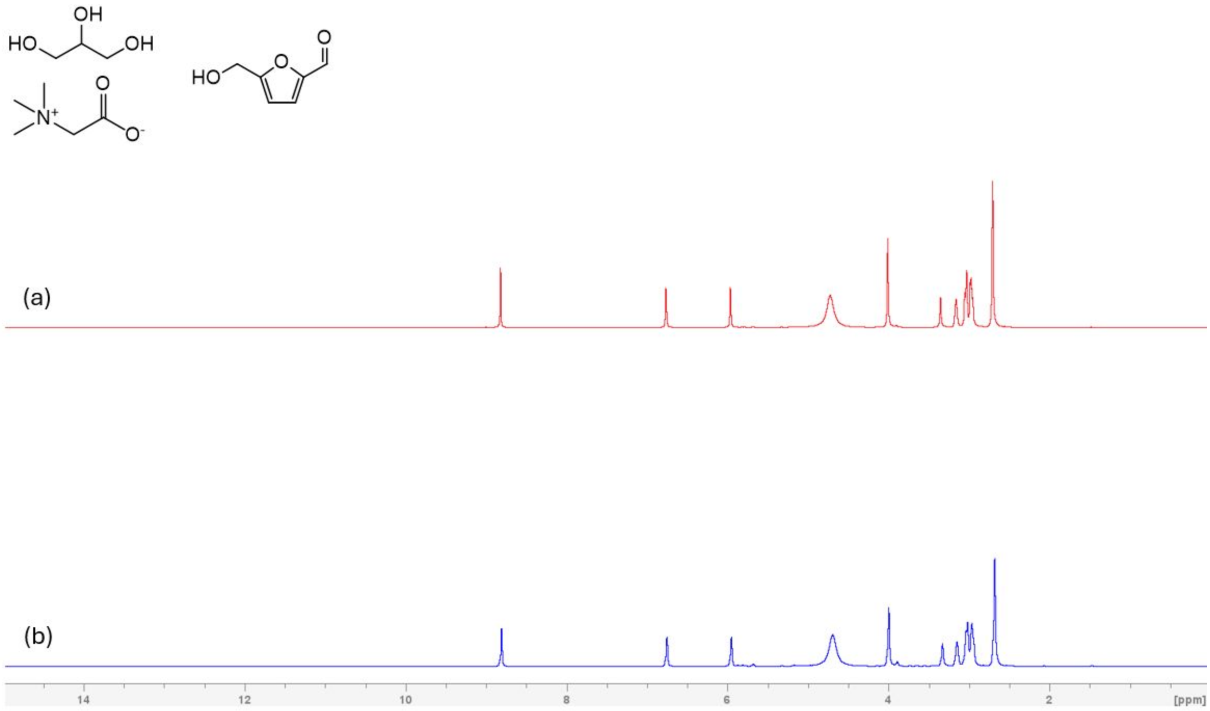

Figure S.16 ^{1}H-NMR in DMSO-d6 (coaxial inset) of Bet-Gly-HMF mixture (a) freshly prepared mixture and (b) same mixture after 30 days

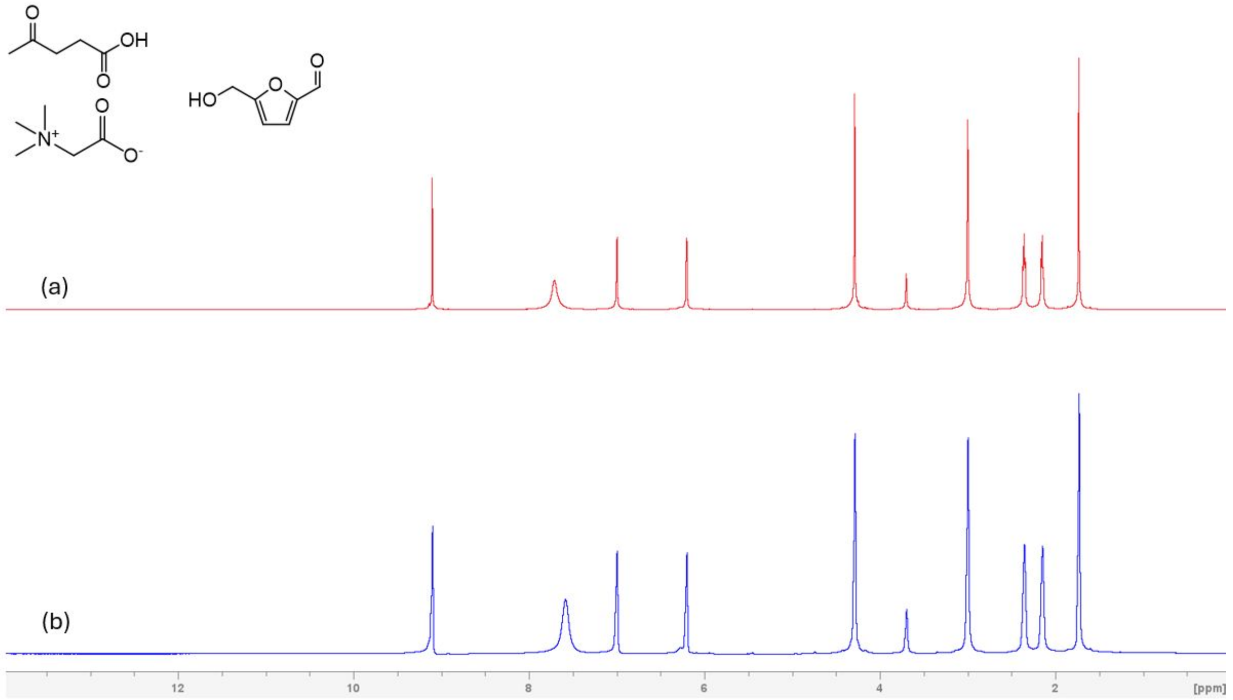

Figure S.17 ^{1}H-NMR in DMSO-d6 (coaxial inset) of Bet-LA-HMF mixture (a) freshly prepared mixture and (b) same mixture after 30 days

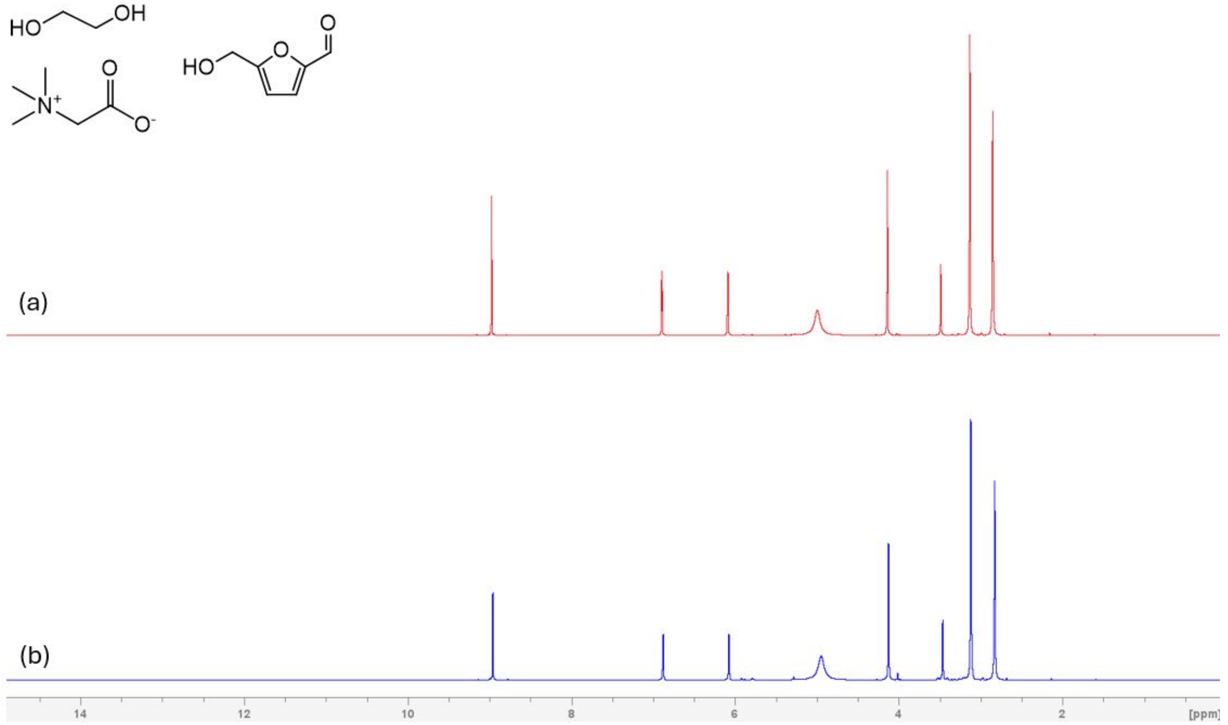

Figure S.18 ^{1}H-NMR in DMSO-d6 (coaxial inset) of Bet-EG-HMF mixture (a) freshly prepared mixture and (b) same mixture after 30 days

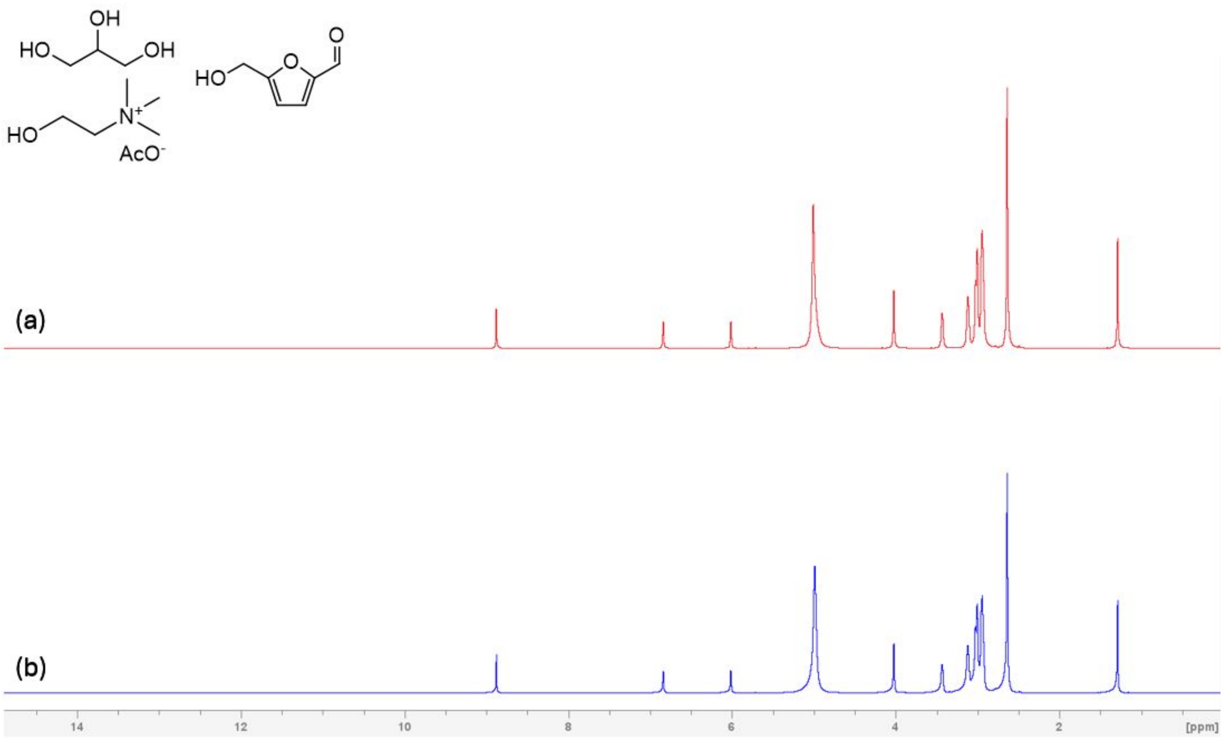

Figure S.19 ^{1}H-NMR in DMSO-d6 (coaxial inset) of ChAc-Gly-HMF mixture (a) freshly prepared mixture and (b) same mixture after 30 days

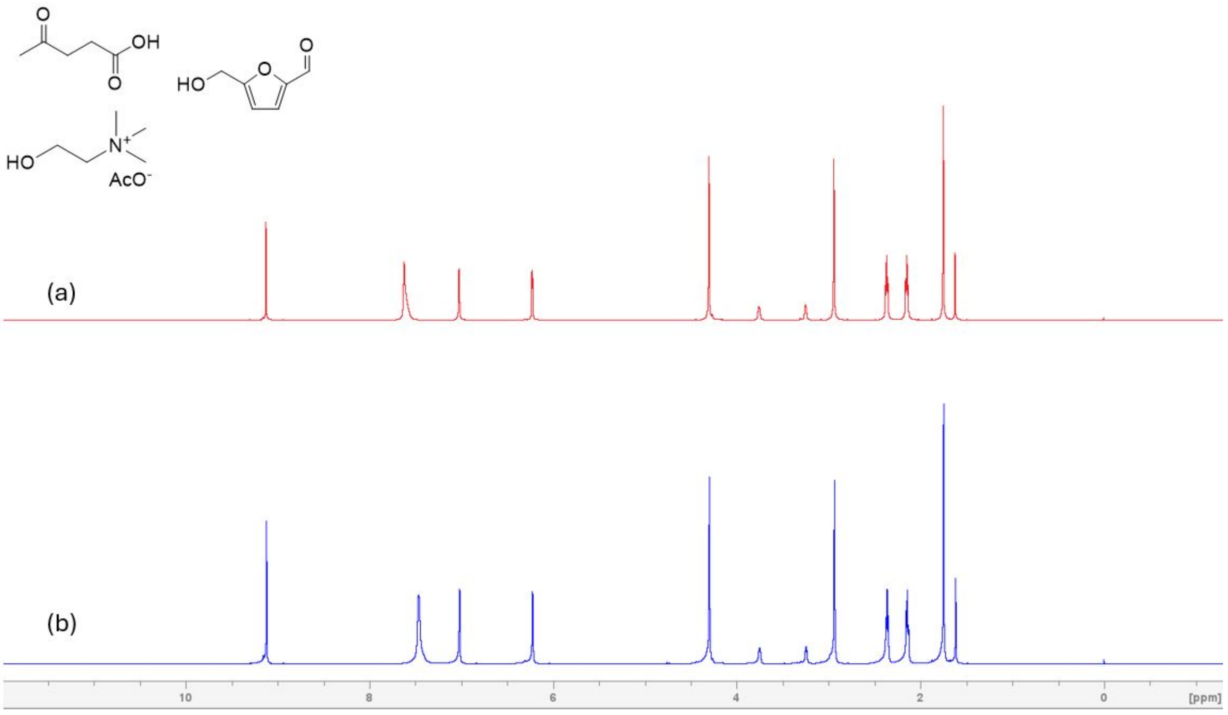

Figure S.20 ^{1}H-NMR in DMSO-d6 (coaxial inset) of ChAc-LA-HMF mixture (a) freshly prepared mixture and (b) same mixture after 30 days

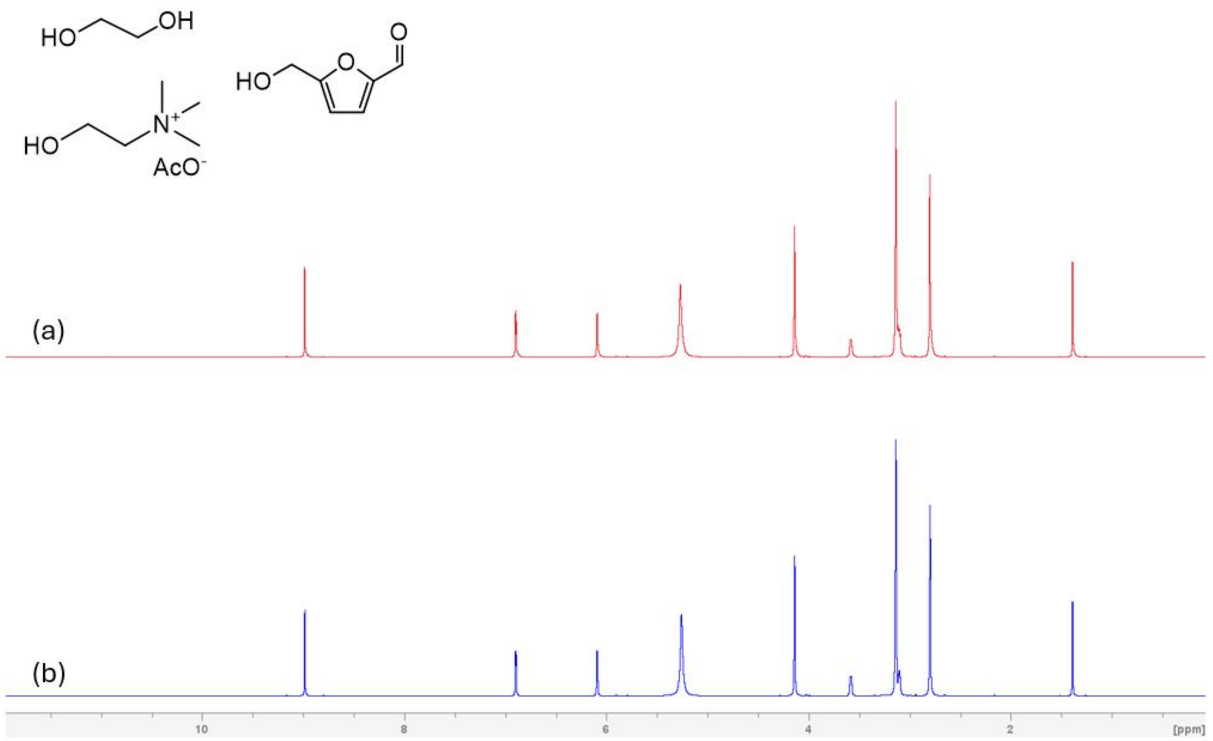

Figure S.21 ^{1}H-NMR in DMSO-d6 (coaxial inset) of ChAc-EG-HMF mixture (a) freshly prepared mixture and (b) same mixture after 30 days

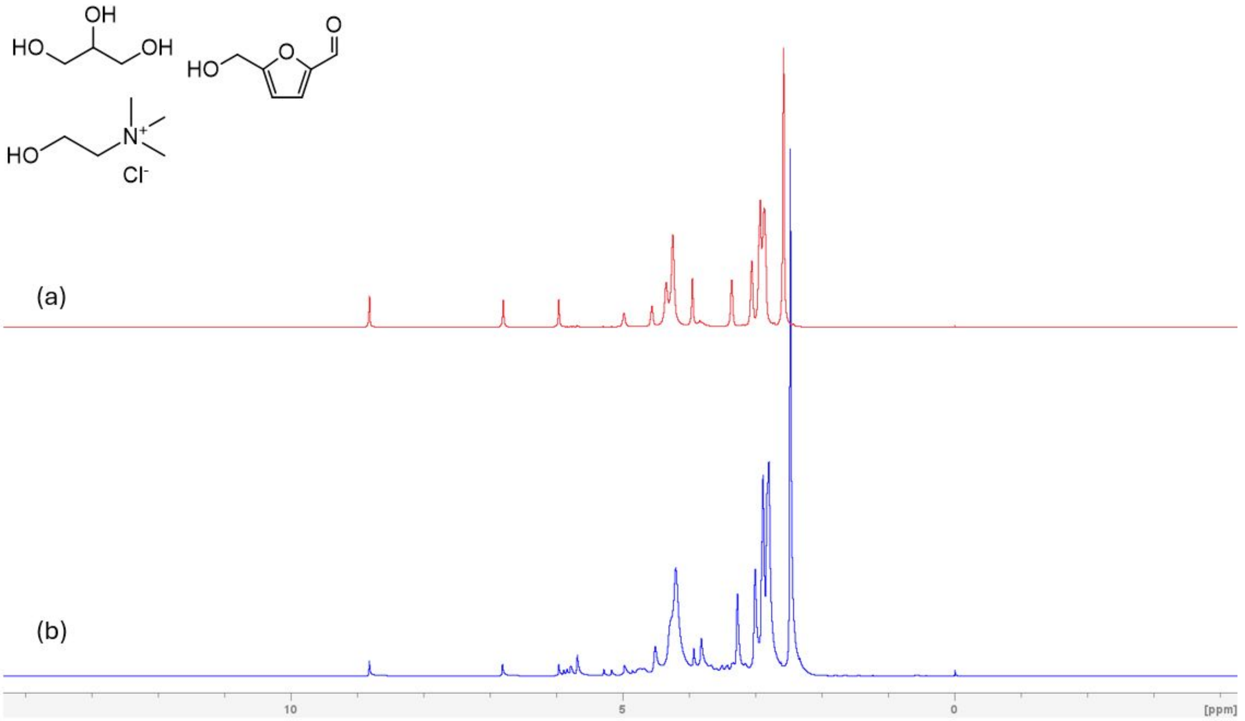

Figure S.22 ^{1}H-NMR in DMSO-d6 (coaxial inset) of ChCl-Gly-HMF mixture (a) freshly prepared mixture and (b) same mixture after 3 days

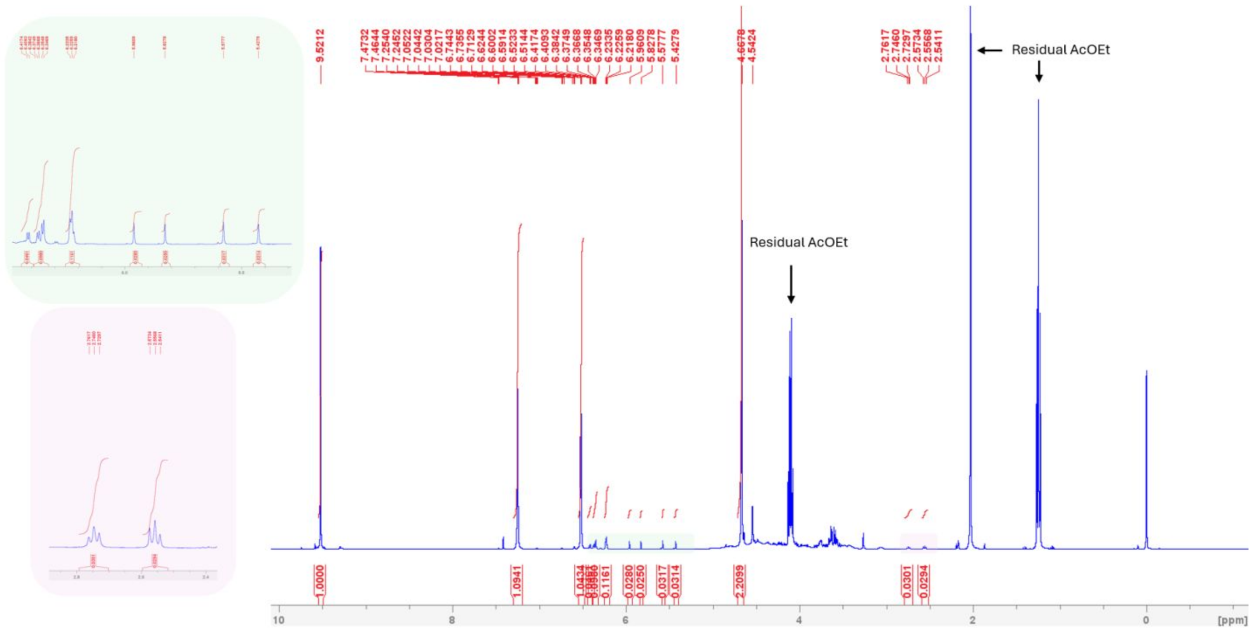

Figure S.23 ^{1}H-NMR, 400 MHz, in $CDCl_3$ of the ChCl-Gly-HMF mixture after extraction

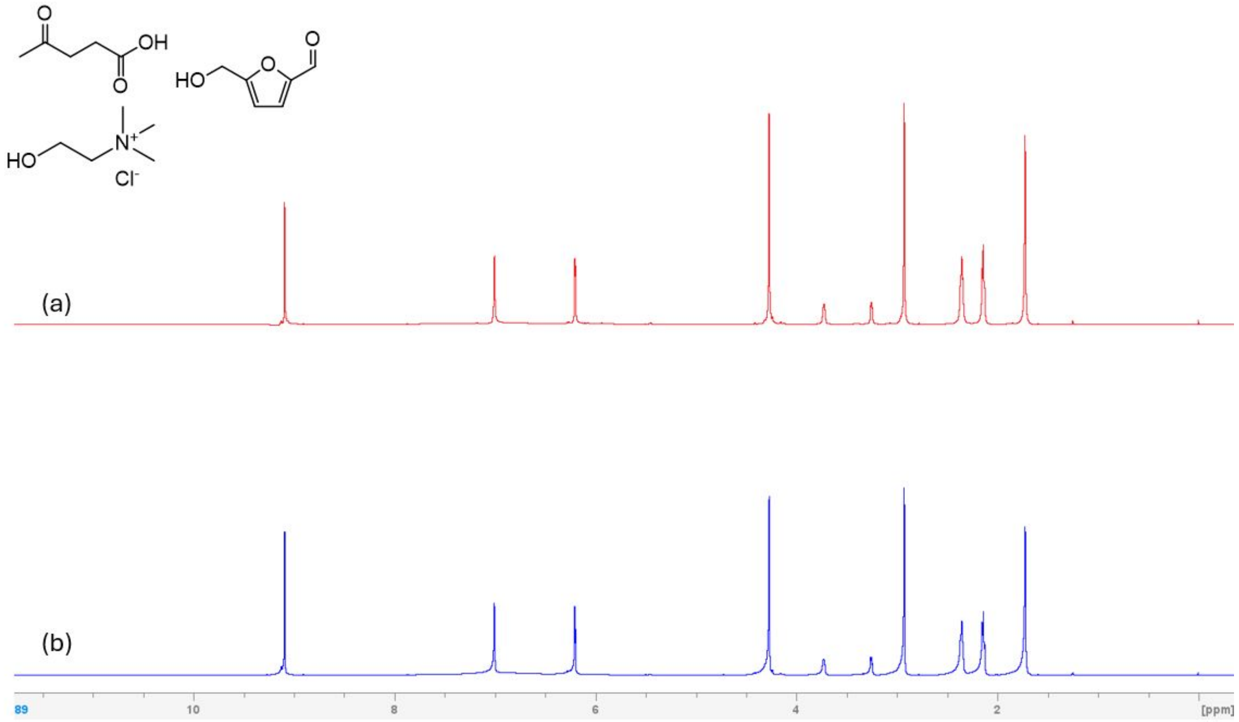

Figure S.24 ^{1}H-NMR in DMSO-d6 (coaxial inset) of ChCl-LA-HMF mixture (a) freshly prepared mixture and (b) same mixture after 30 days

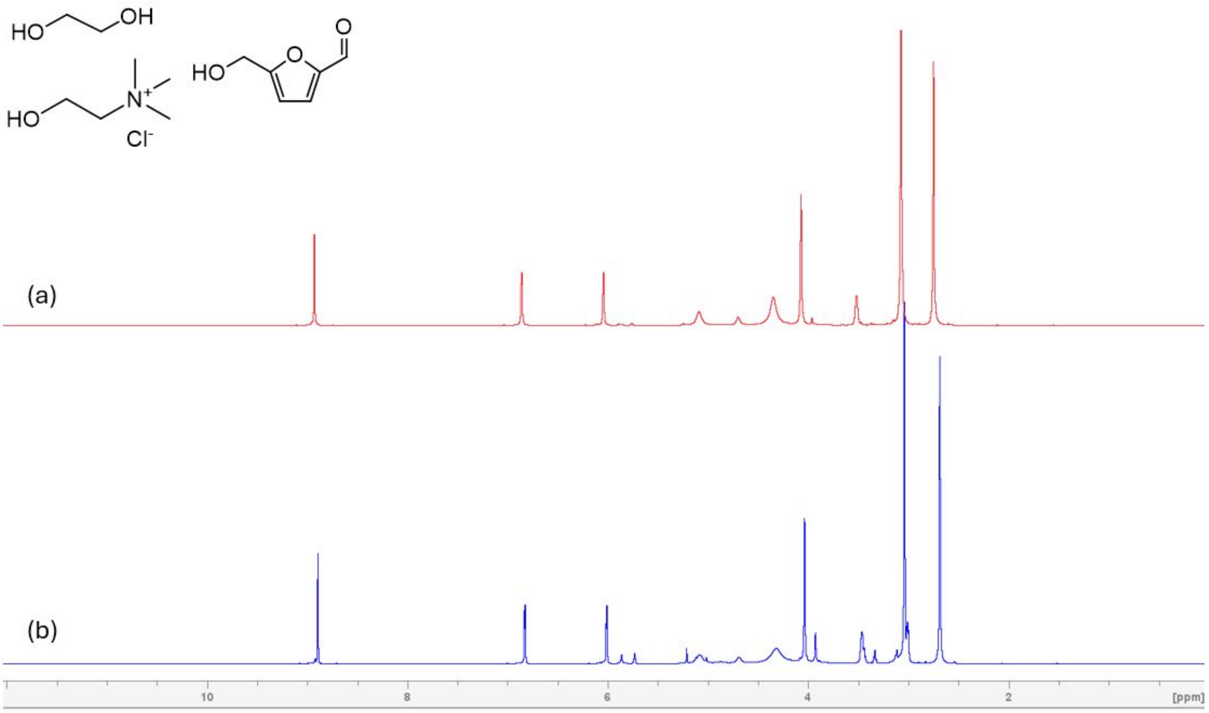

Figure S.25 ^{1}H-NMR in DMSO-d6 (coaxial inset) of ChCl-EG-HMF mixture (a) freshly prepared mixture and (b) same mixture after 30 days

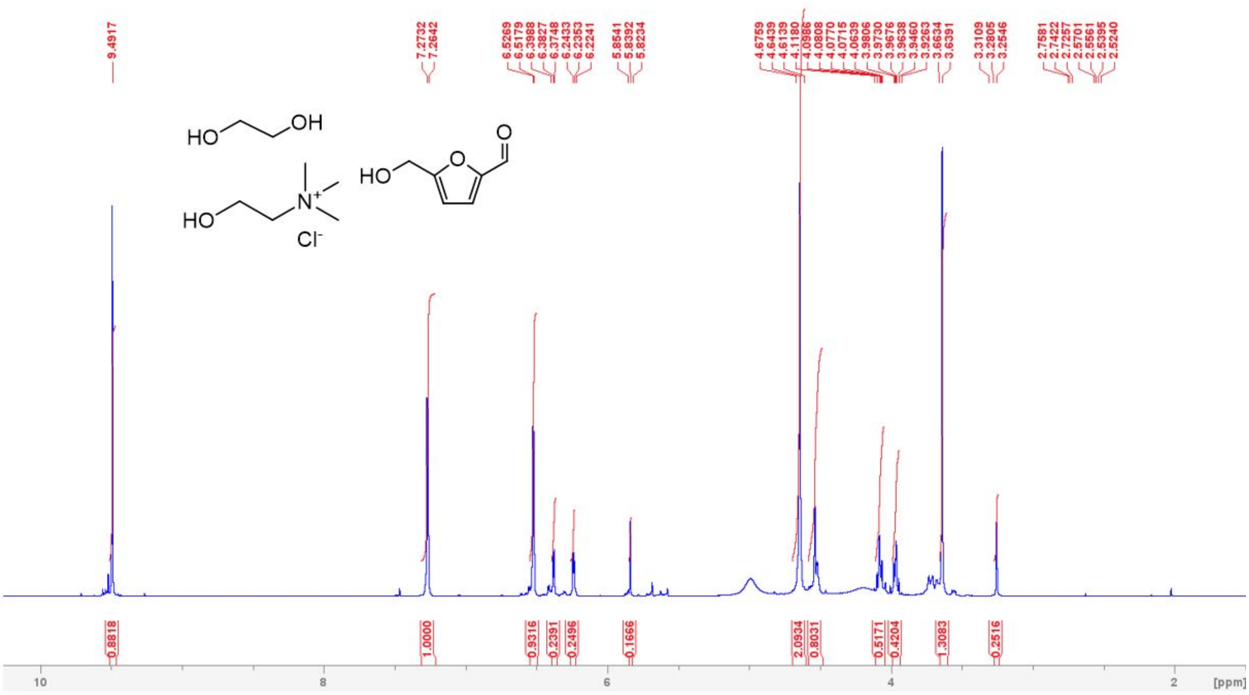

Figure S.26 ^{1}H-NMR, 400 MHz, in $CDCl_3$ of the ChCl-EG-HMF mixture after extraction

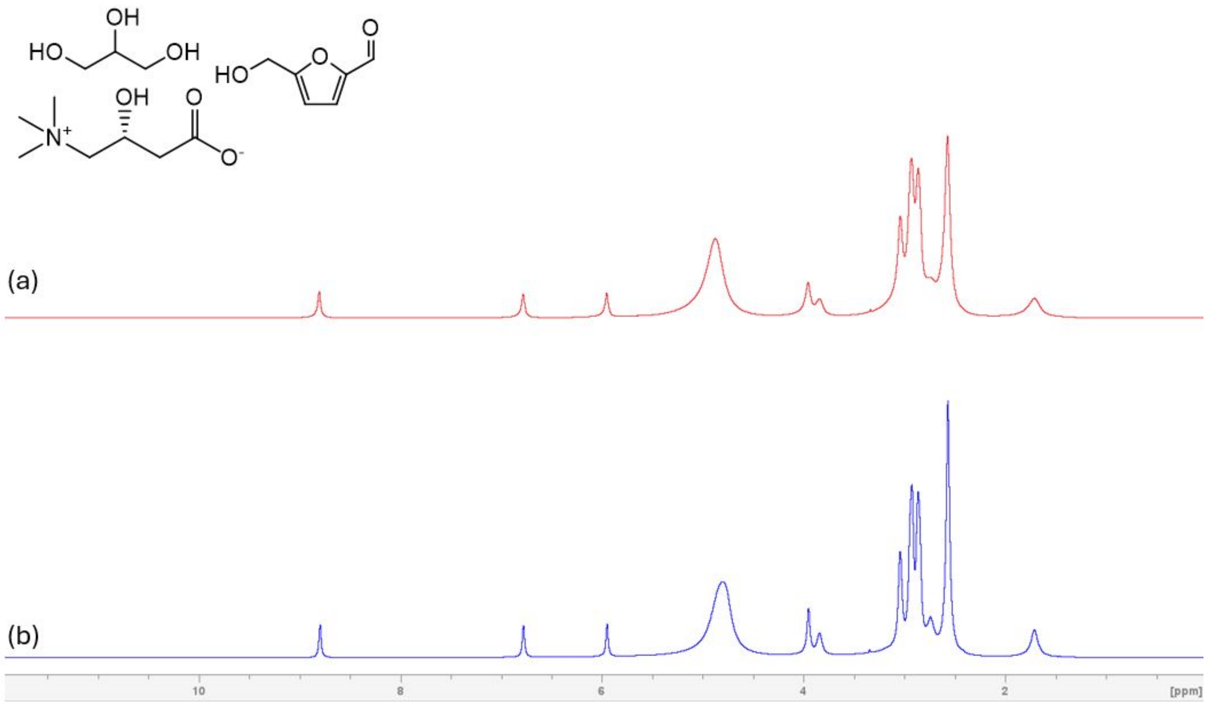

Figure S.27 ^{1}H-NMR in DMSO-d6 (coaxial inset) of Carn-Gly-HMF mixture (a) freshly prepared mixture and (b) same mixture after 30 days

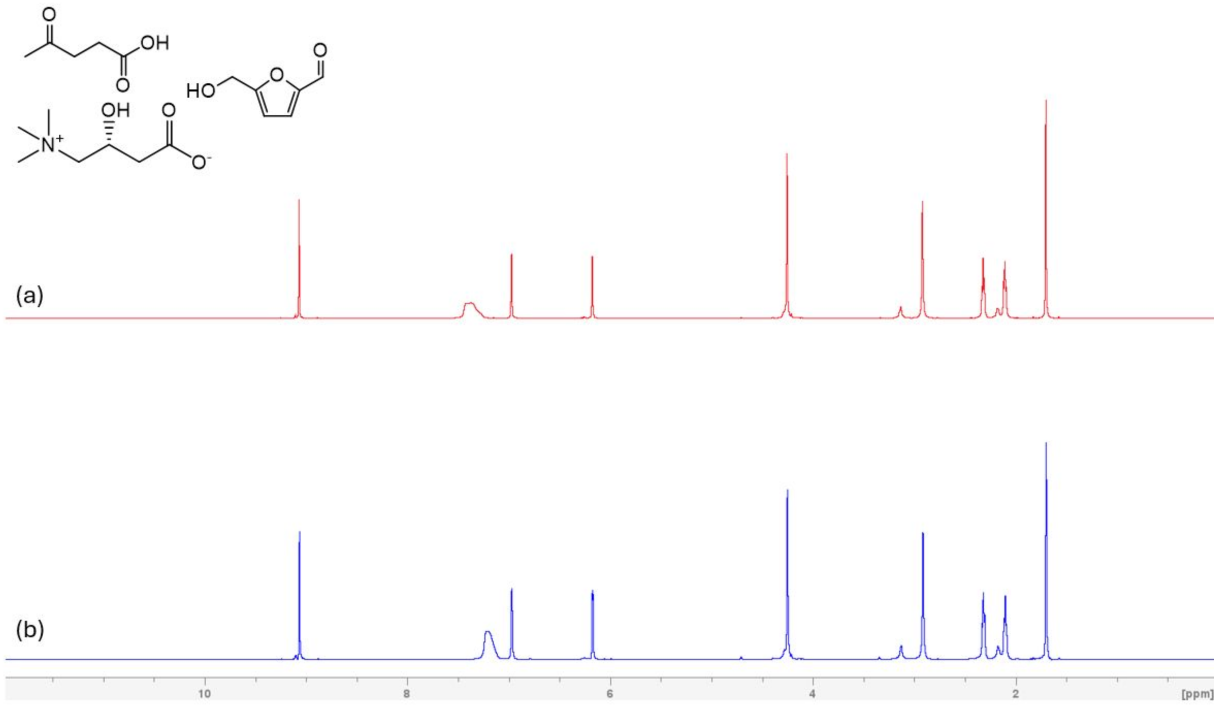

Figure S.28 ^{1}H-NMR in DMSO-d6 (coaxial inset) of Carn-LA-HMF mixture (a) freshly prepared mixture and (b) same mixture after 30 days

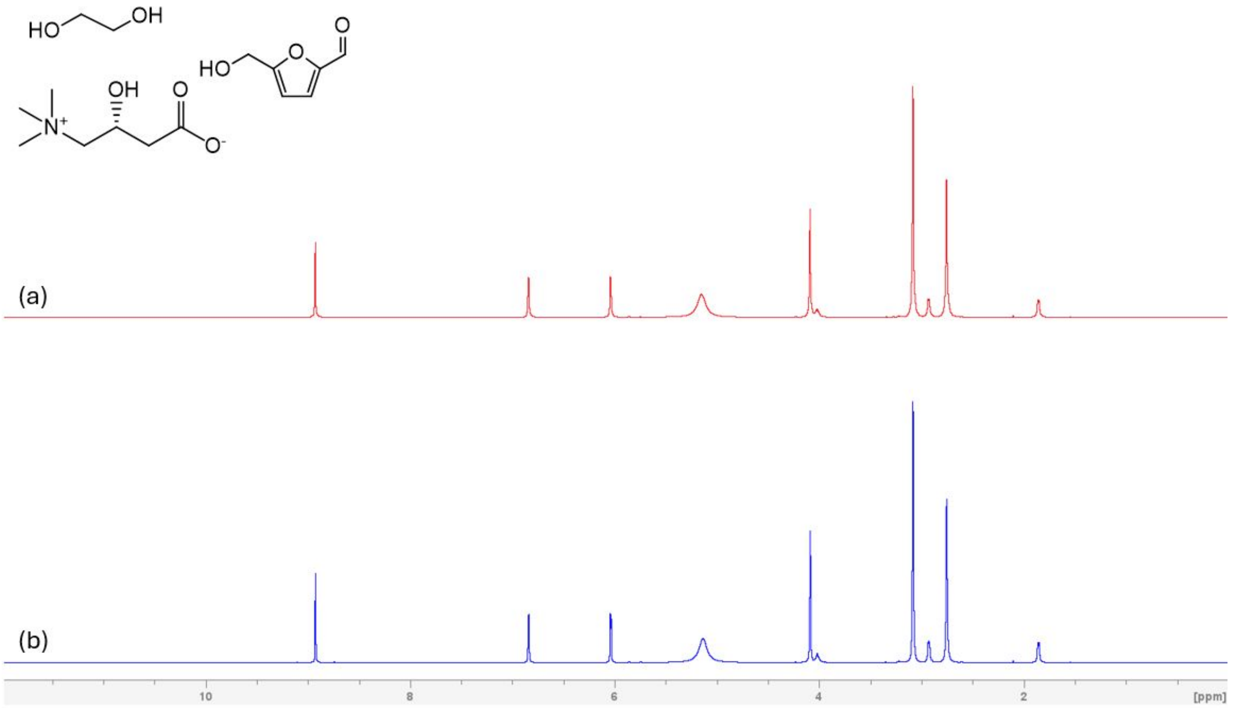

Figure S.29 ^{1}H-NMR in DMSO-d6 (coaxial inset) of Carn-EG-HMF mixture (a) freshly prepared mixture and (b) same mixture after 30 days

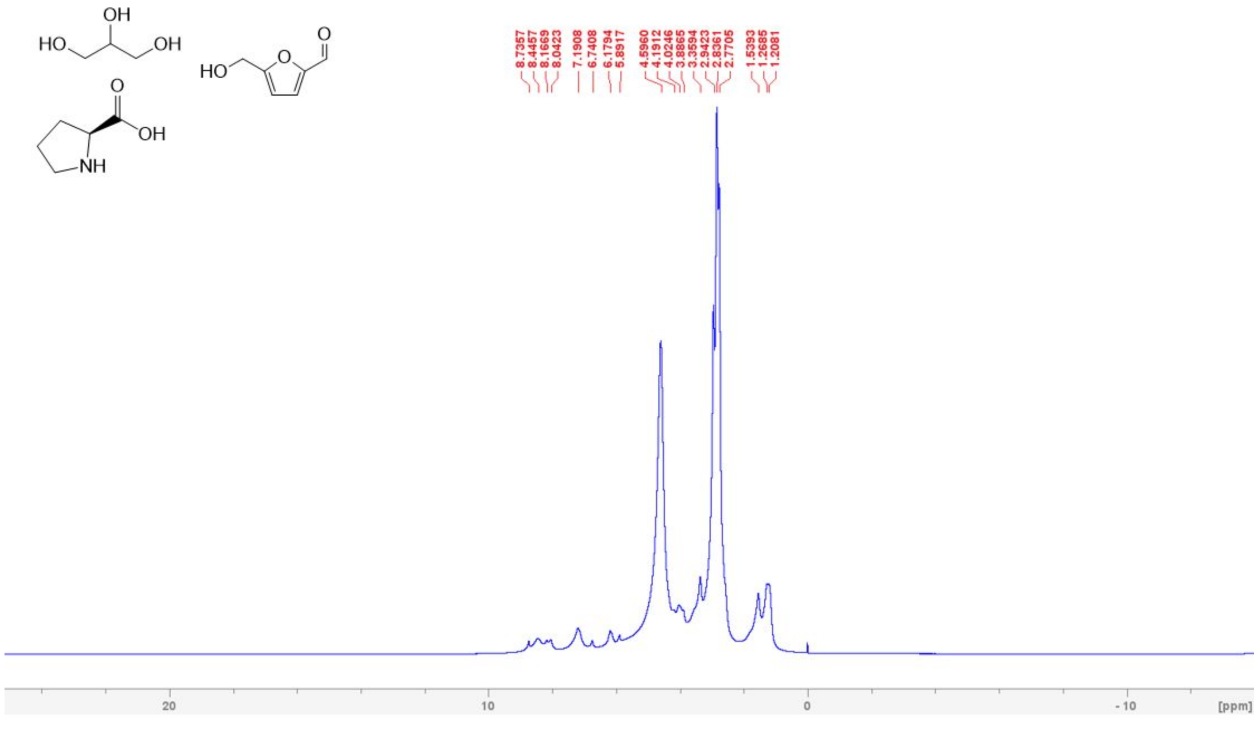

Figure S.30 ^{1}H-NMR in DMSO-d6 (coaxial inset) of Pro-Gly-HMF mixture

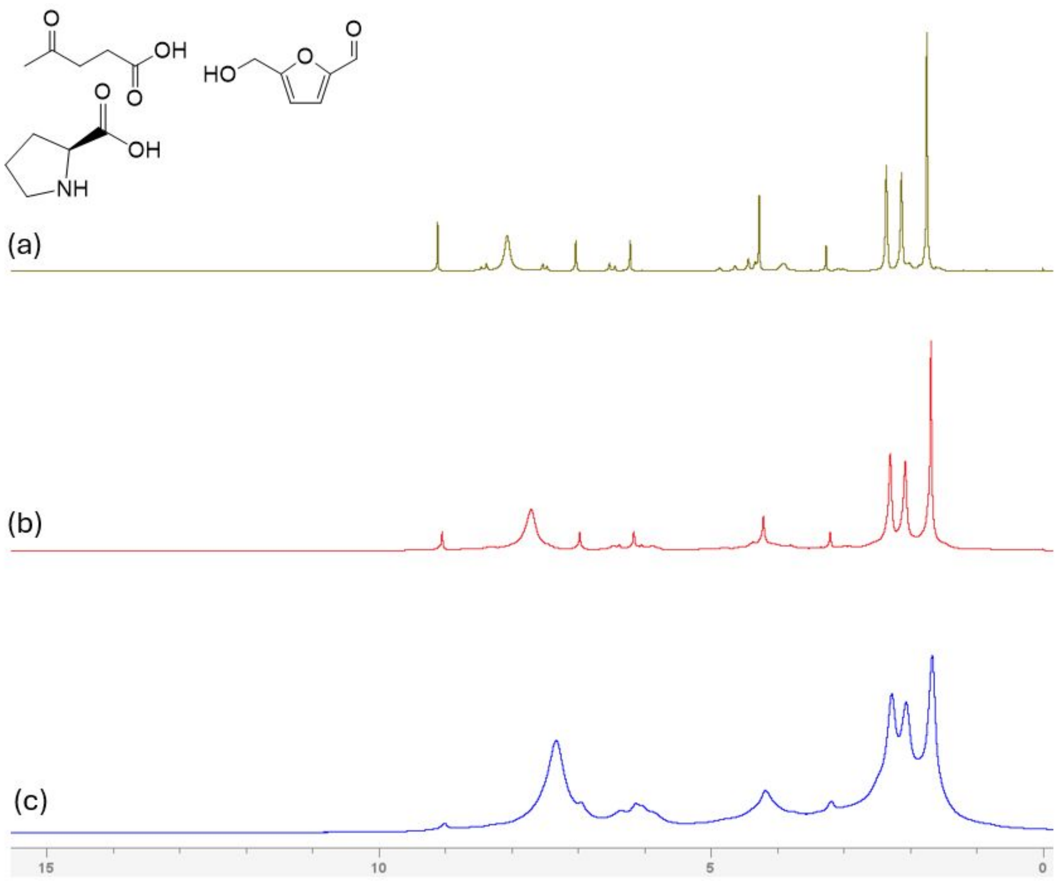

Figure S.31 ^{1}H-NMR in DMSO-d6 (coaxial inset) of Pro-LA-HMF mixture (a) freshly prepared mixture, (b) same mixture after 5 days and (c) same mixture after 30 days

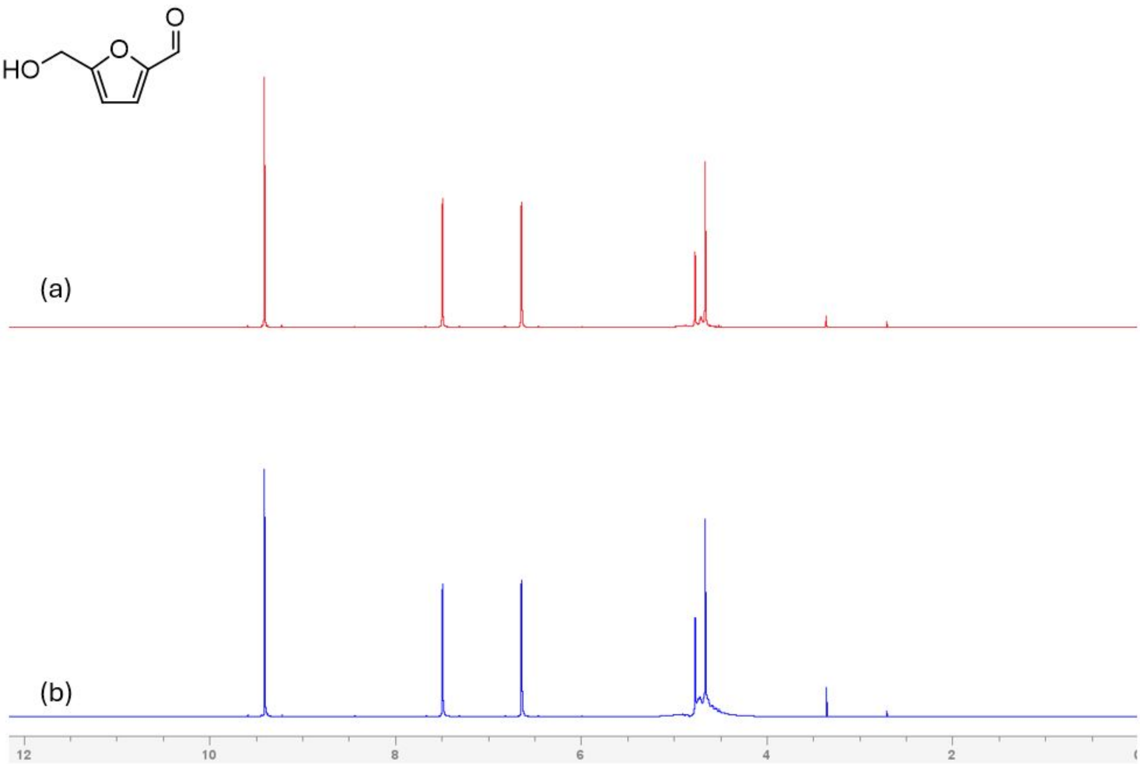

Figure S.32 ^{1}H-NMR in D_2O of pure HMF (a) freshly prepared, (b) same sample after 30 days

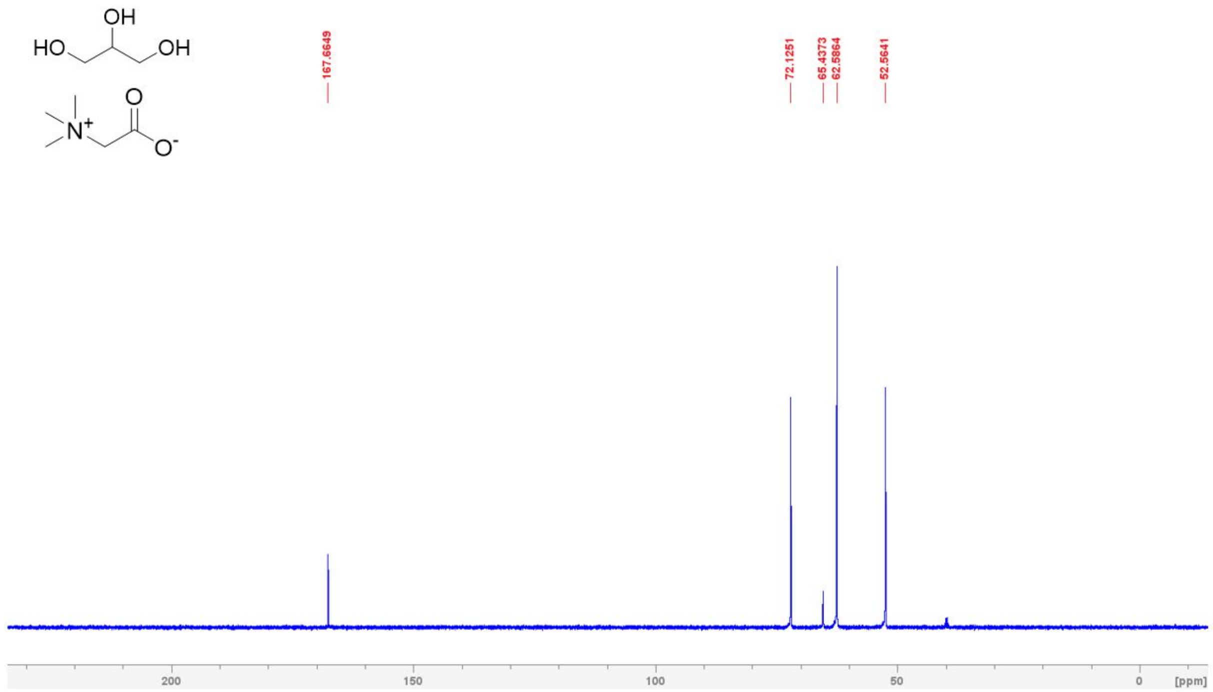

Figure S.33 ^{13}C-NMR in DMSO-d6 (coaxial inset) of Bet-Gly mixture

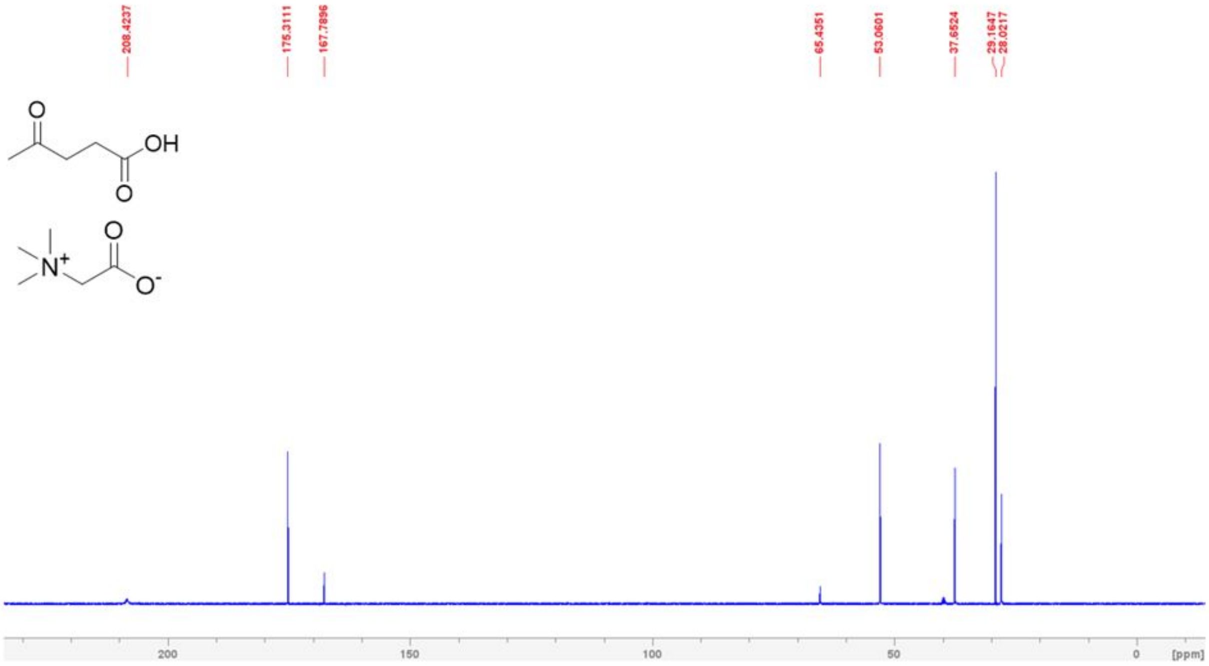

Figure S.34 ^{13}C-NMR in DMSO-d6 (coaxial inset) of Bet-LA mixture

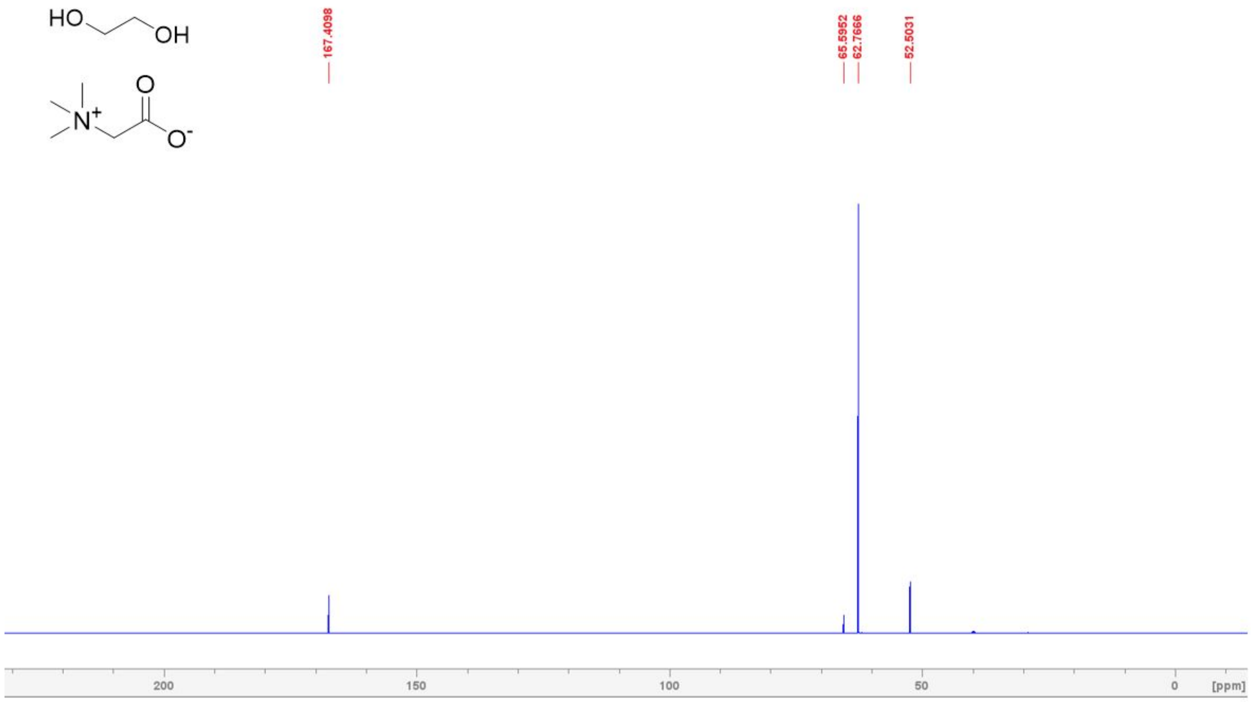

Figure S.35 ^{13}C-NMR in DMSO-d6 (coaxial inset) of Bet-EG mixture

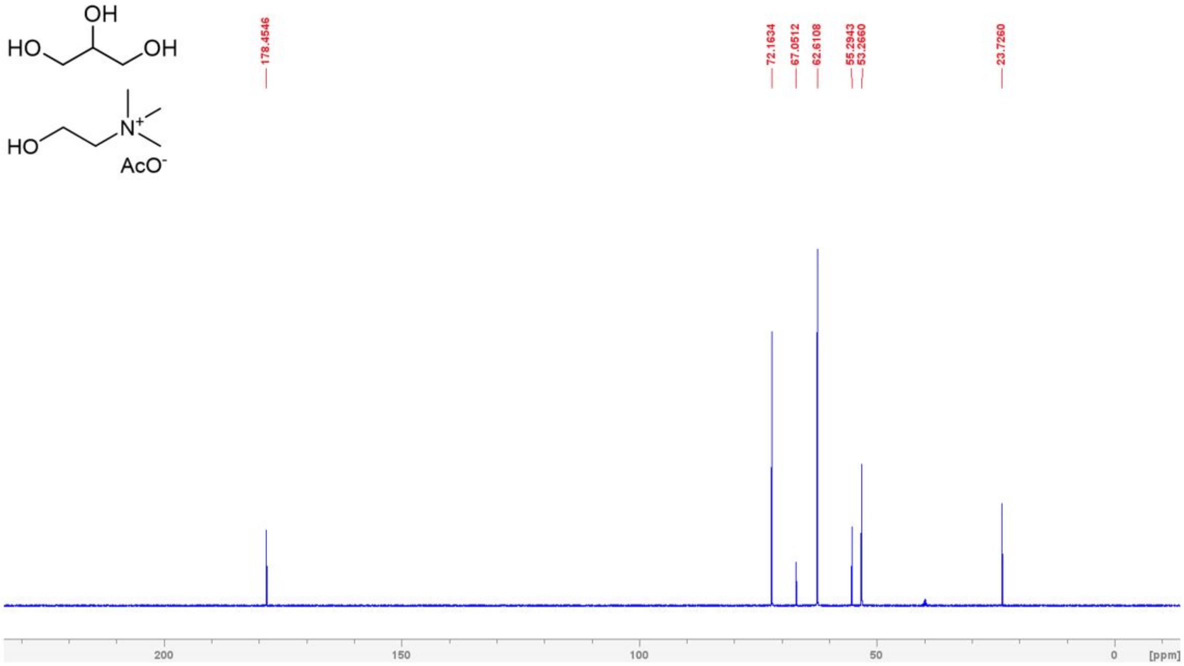

Figure S.36 ^{13}C-NMR in DMSO-d6 (coaxial inset) of ChOAc-Gly mixture

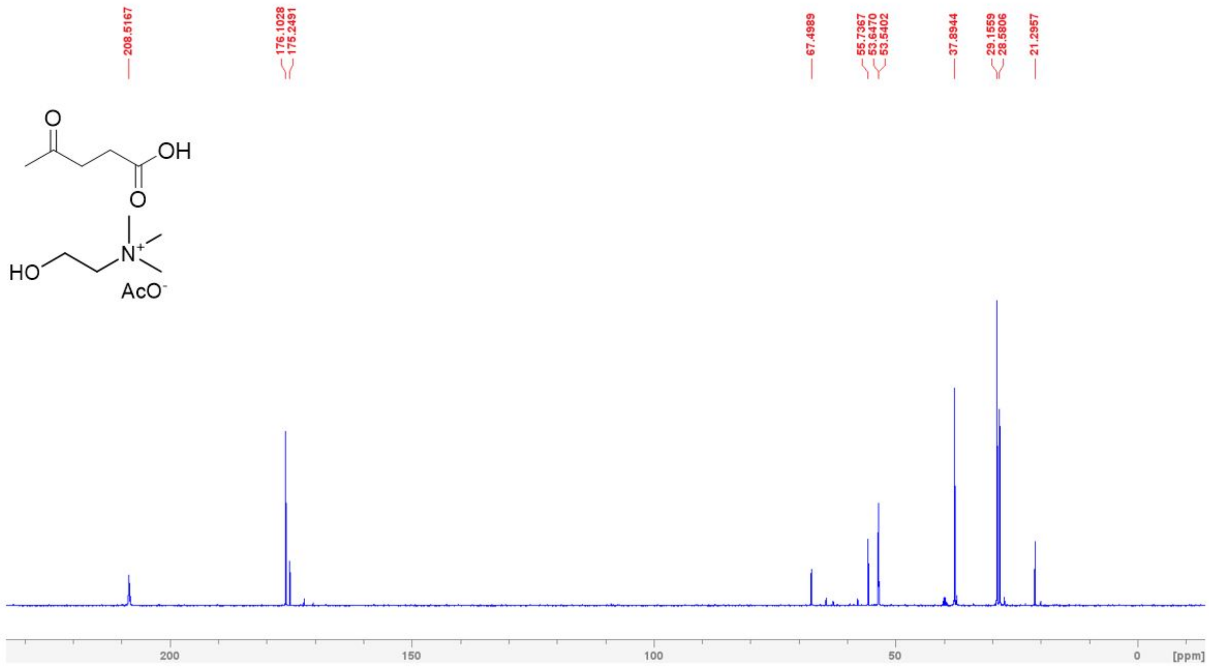

Figure S.37 ^{13}C-NMR in DMSO-d6 (coaxial inset) of ChOAc-LA mixture

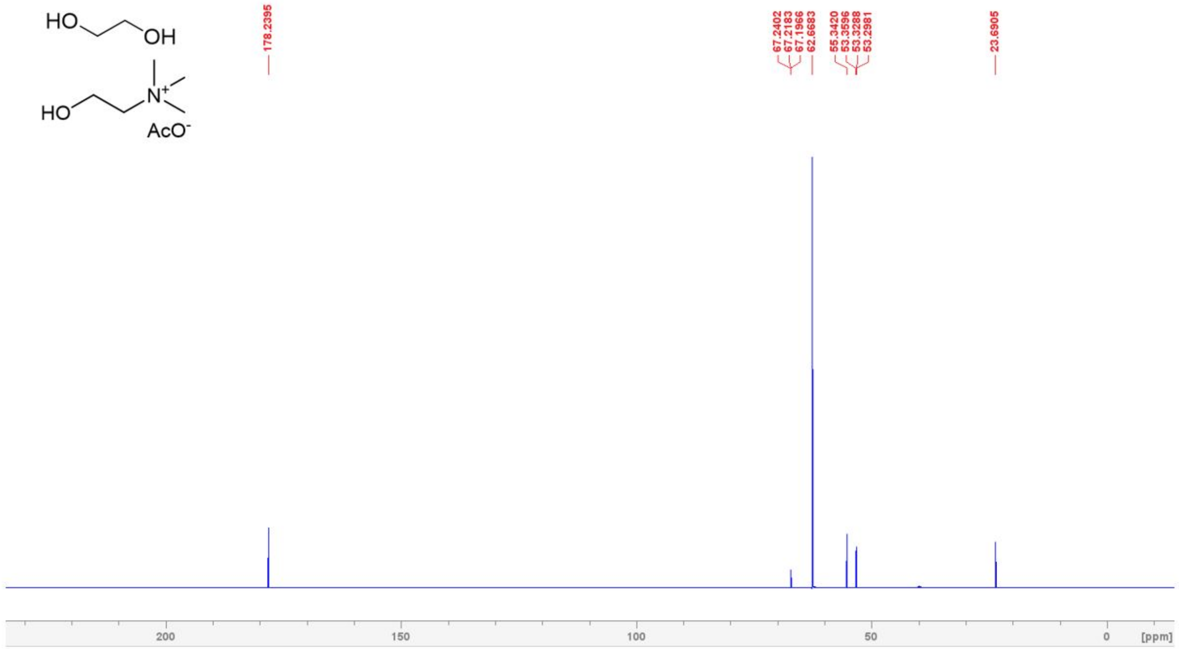

Figure S.38 ^{13}C-NMR in DMSO-d6 (coaxial inset) of ChOAc-EG mixture

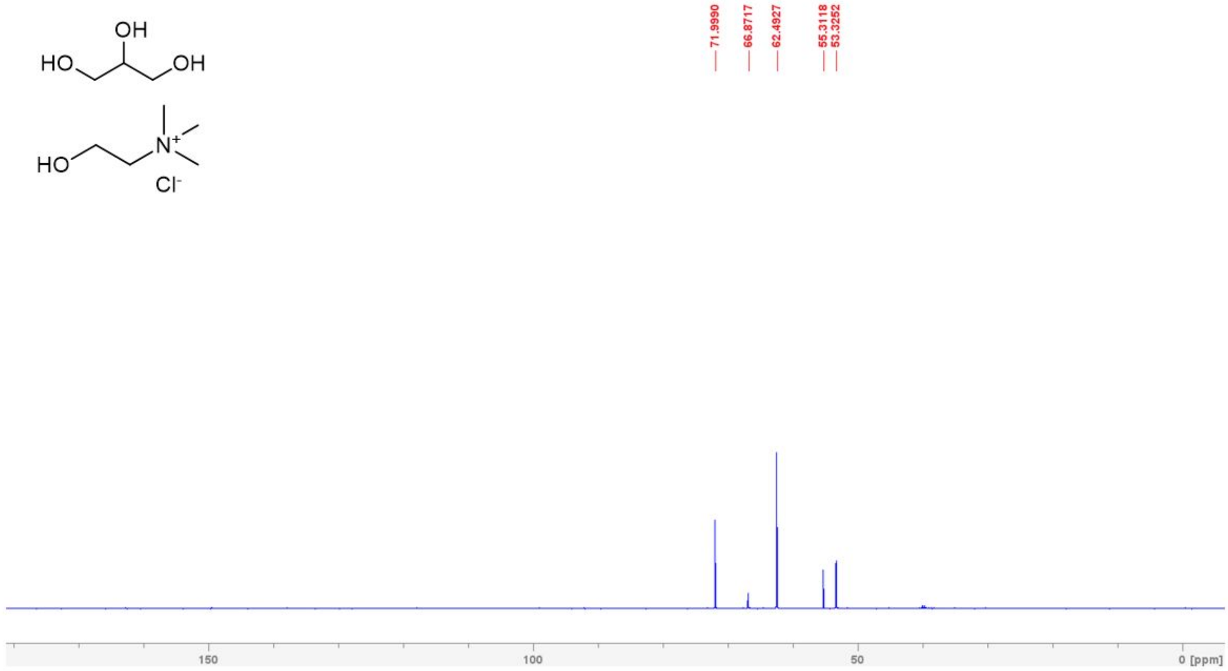

Figure S. 39 ^{13}C-NMR in DMSO-d6 (coaxial inset) of ChCl-Gly mixture

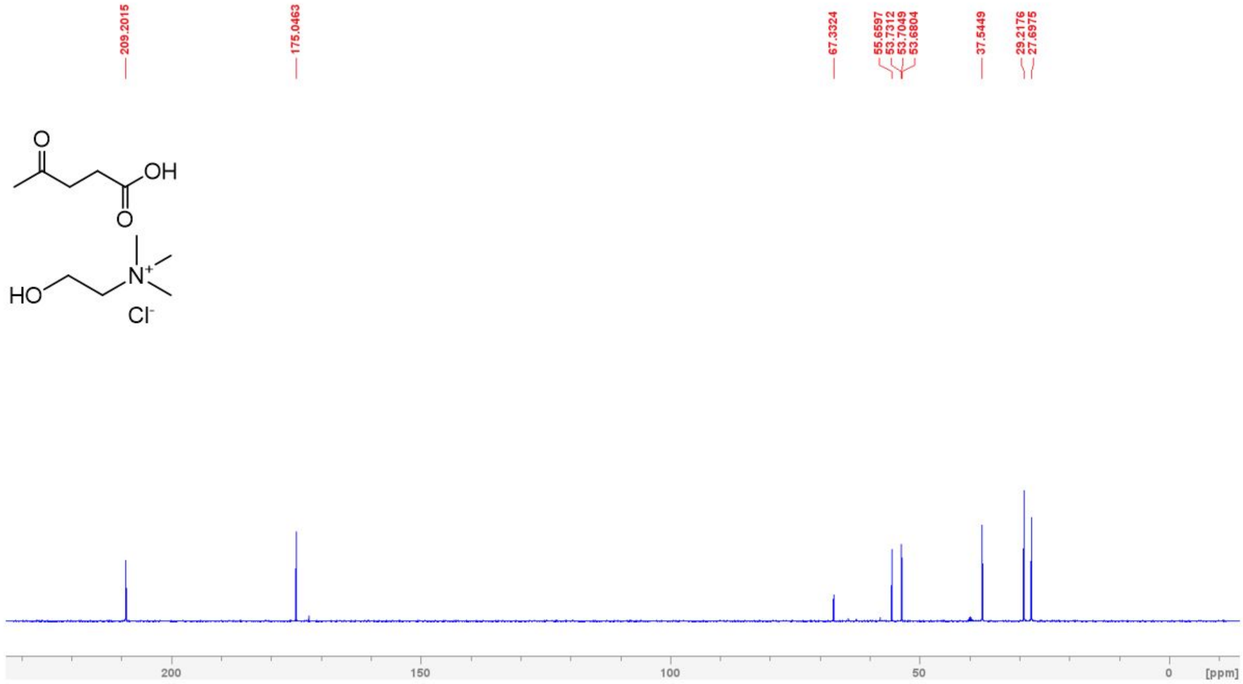

Figure S. 40 ^{13}C-NMR in DMSO-d6 (coaxial inset) of ChCl-LA mixture

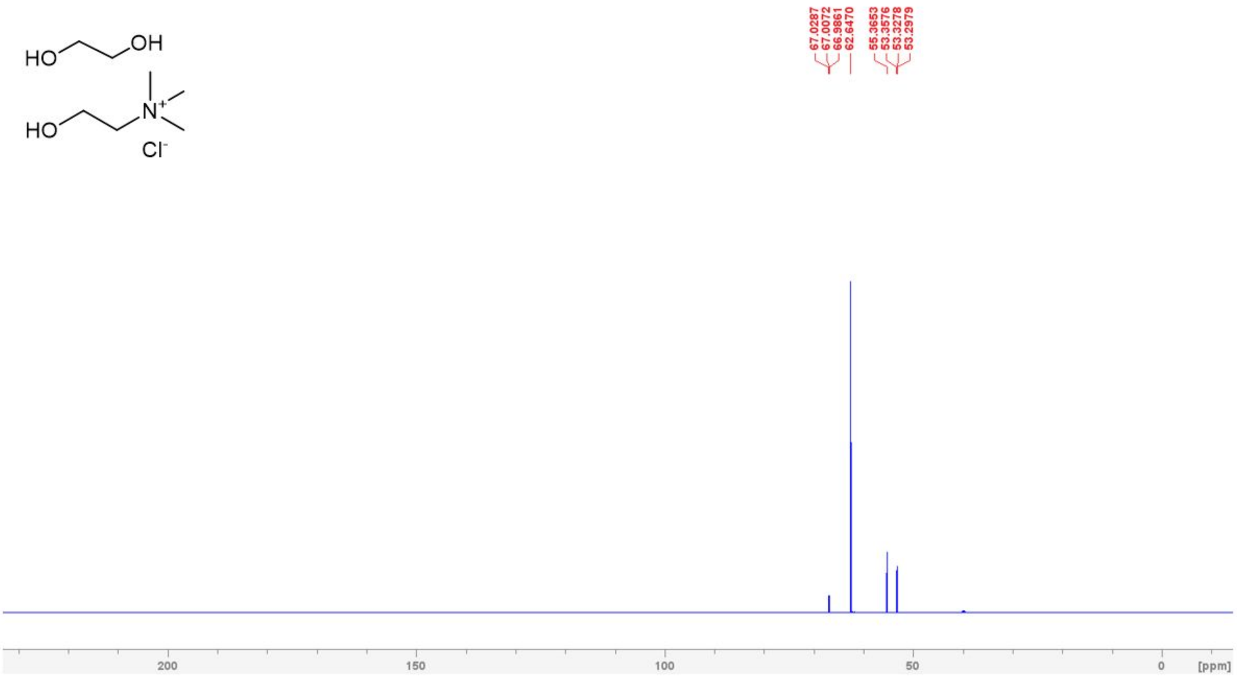

Figure S. 41 ^{13}C-NMR in DMSO-d6 (coaxial inset) of ChCl-EG mixture

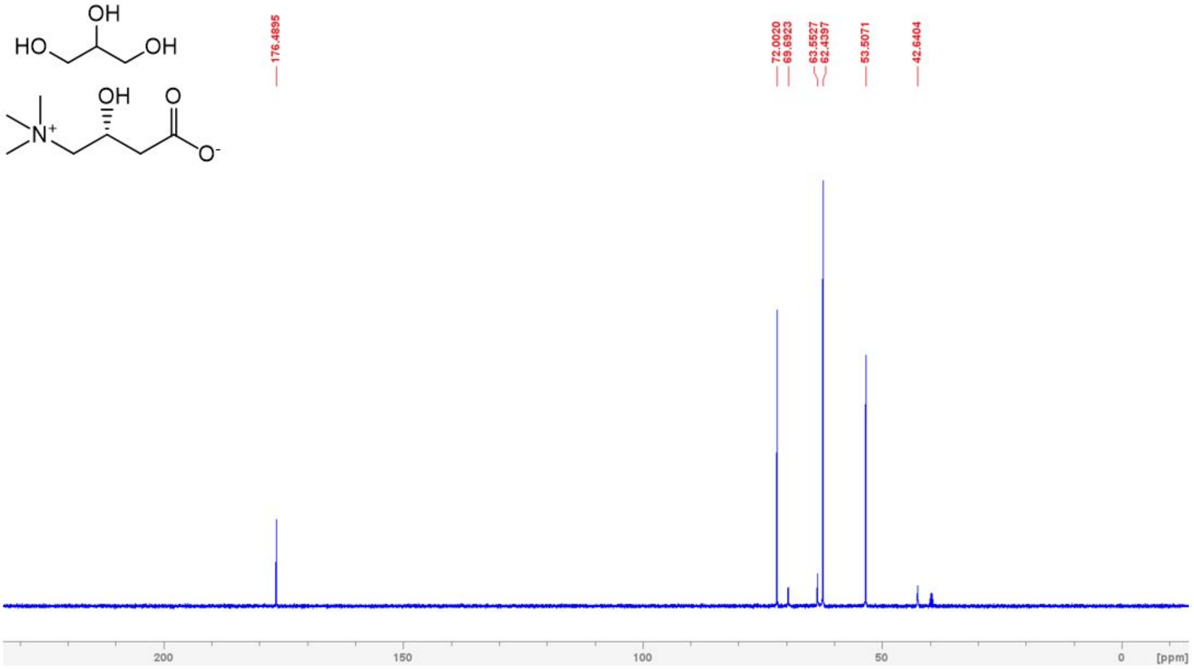

Figure S. 42 ^{13}C-NMR in DMSO-d6 (coaxial inset) of Carnitine-Gly mixture

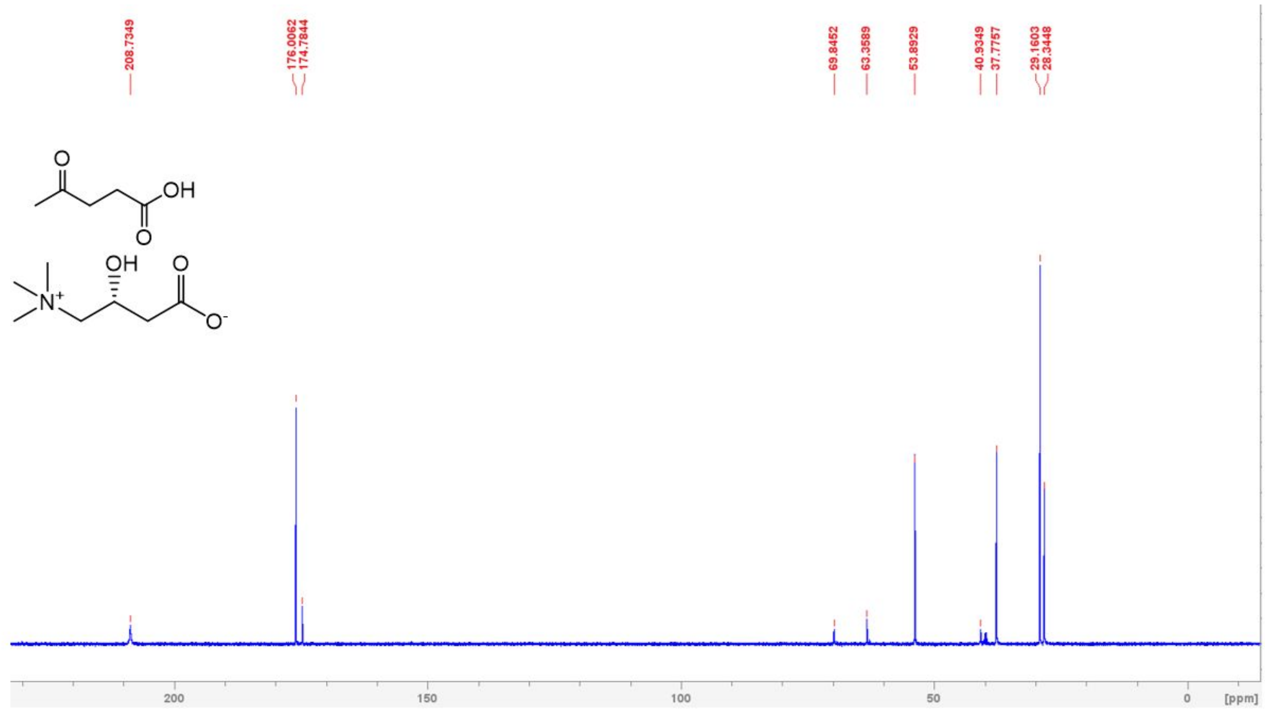

Figure S. 43 ^{13}C-NMR in DMSO-d6 (coaxial inset) of Carnitine-LA mixture

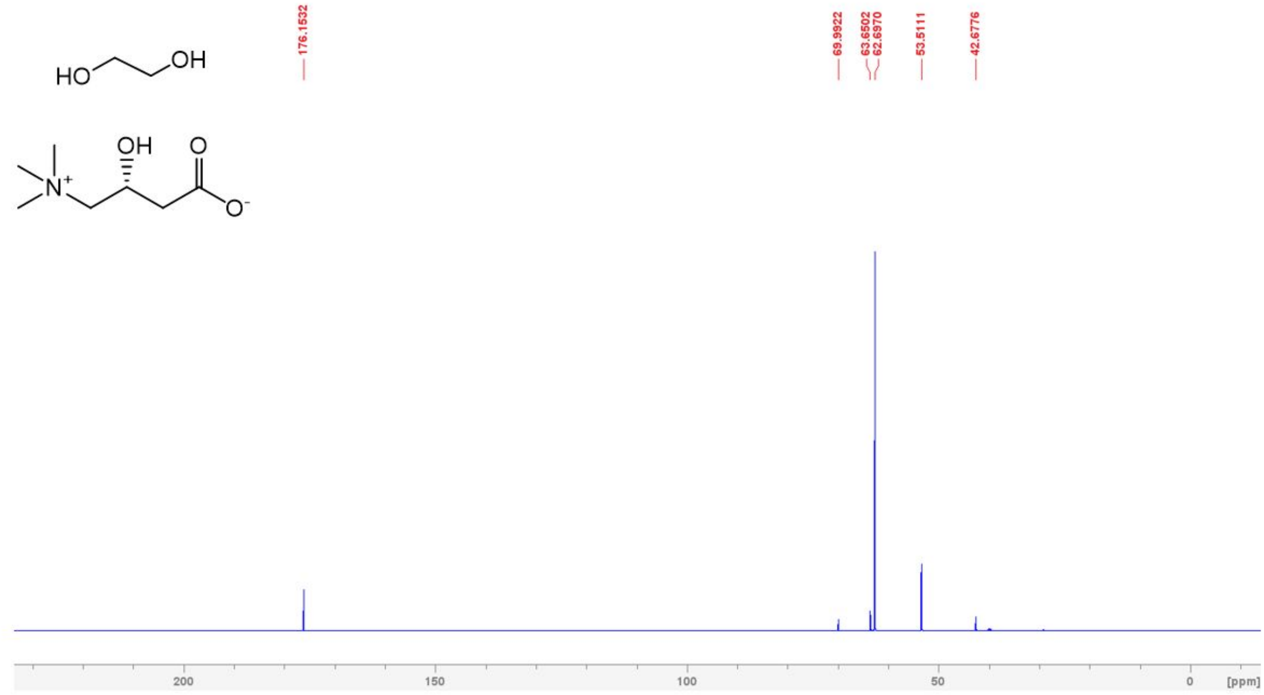

Figure S. 44 ^{13}C-NMR in DMSO-d6 (coaxial inset) of Carnitine-EG mixture

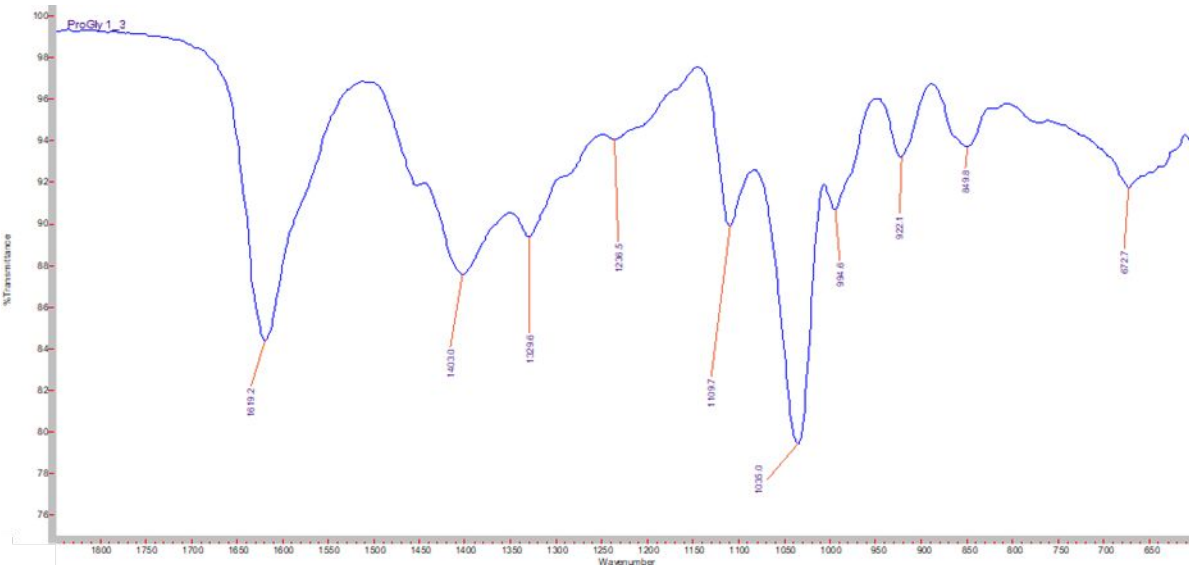

Figure S. 45. Expansion of FT-IR spectrum of Pro-Gly.

Table S. 1 ^{13}C NMR chemical shift of carbonyl/carboxyl/carboxylate signals in pure DES

	δ(^{13}C=O), ppm				
**DES**	**Bet**	**LA**	**Ac**	**Car**	**Pro**
Bet-Gly	167.6	-	-	-	-
Bet-LA	167.8	208.4; 175.3			-
Bet-EG	167.4	-	-	-	-
ChCl-Gly	-	-	-	-	-
ChCl-LA		209.2; 175.0	-	-	-
ChCl-EG	-	-	-	-	-
ChAc-Gly	-	-	178.5	-	-
ChAc-LA		208.5; 176.1	175.2	-	-
ChAc-EG	-	-	178.2	-	-
Carn-Gly	-	-	-	176.5	-
Carn-LA		208.7; 176.0		174.8	-
Carn-EG	-	-	-	176.2	-
Pro-Gly	-				173.3
Pro-LA	-	208.9; 175.9			173.5
Pro-EG					

Table S.2 EcoScale evaluation for the prepared DESs – pt1

The penalty points to calculate the EcoScale			*Betaine:Gycerol*	*Betaine:Lev. Ac.*	*Betaine:EG*	*ChCl:Glycerol*	*ChCl:Lev Acid*	*ChCl:EG*	*ChOAc:Glycerol*	*ChOAc:Lev Acid*	*ChOAc:EG*
	**Parameter**	**Penalty points**									
**1**	**Yield**	(100 – %yield)/2									
**2**	**Price of reaction components (to obtain 10mmol of end product)**										
	Inexpensive (< $10)	0	0	0	0	0	0	0	0	0	0
	Expensive (> $10 and < $50)	3									
	Very expensive (> $50)	5									
**3**	**Safety (Based on the hazard warning symbols)**										
	N (dangerous for environment)	5									
	T (toxic)	5		5	5		5	5		5	5
	F (highly flammable)	5									
	E (explosive)	10									
	F+ (extremely flammable)	10									
	T+ (extremely toxic)	10									
**4**	**Technical setup**										
	Common setup	0	0	0	0						
	Instruments for controlled addition of chemicals (Dropping funnel, syringe pump, gas pressure regulator, etc)	1									
	Unconventional activation technique (Microwave irradiation, ultrasound or photochemical activation, etc.)	2									
	Pressure equipment, > 1 atm ($scCO_2$, high pressure hydrogenation equipment, etc.)	3									
	Any additional special glassware	1									
	(Inert) gas atmosphere	1									
	Glove box	3									
**5**	**Temperature/time**										
	Room temperature, < 1 h	0									
	Room temperature, < 24 h	1									
	Heating, < 1 h	2	2	2	2	2	2	2	2	2	2
	Heating, > 1 h	3									
	Cooling to 0°C	4									
	Cooling, < 0°C	5									
**6**	**Workup and purification**										
	None	0	0	0	0						
	Cooling to room temperature	0									
	Adding solvent	0									
	Simple filtration	0									
	Removal of solvent with bp < 150°C	0									
	Crystallization and filtration	1									
	Removal of solvent with bp > 150°C	2									
	Solid phase extraction	2									
	Distillation	3									
	Sublimation	3									
	Liquid-liquid extraction (If applicable, the process includes drying of solvent with desiccant and filtration of desiccant.)	3									
	Classical chromatography	10									
		**Sum penalty points**	2	7	7	2	7	7	2	7	7
		**EcoScale**	98	93	93	98	93	93	98	93	93

Table S.3 EcoScale evaluation for the prepared DESs – pt2

The penalty points to calculate the EcoScale			*Carnitine:Gly*	*Carnitine:Lev Acid*	*Carnitine:EG*	*Proline:Gly*	*Prroline:Lev.Acid*	*Proline:EG*
	**Parameter**	**Penalty points**						
**1**	**Yield**	(100 – %yield)/2						
**2**	**Price of reaction components (to obtain 10mmol of end product)**							
	Inexpensive (< $10)	0				0	0	0
	Expensive (> $10 and < $50)	3	3	3	3			
	Very expensive (> $50)	5						
**3**	**Safety (Based on the hazard warning symbols)**							
	N (dangerous for environment)	5						
	T (toxic)	5		5	5		5	5
	F (highly flammable)	5						
	E (explosive)	10						
	F+ (extremely flammable)	10						
	T+ (extremely toxic)	10						
**4**	**Technical setup**							
	Common setup	0						
	Instruments for controlled addition of chemicals (Dropping funnel, syringe pump, gas pressure regulator, etc)	1						
	Unconventional activation technique (Microwave irradiation, ultrasound or photochemical activation, etc.)	2						
	Pressure equipment, > 1 atm ($scCO_2$, high pressure hydrogenation equipment, etc.)	3						
	Any additional special glassware	1						
	(Inert) gas atmosphere	1						
	Glove box	3						
**5**	**Temperature/time**							
	Room temperature, < 1 h	0						
	Room temperature, < 24 h	1						
	Heating, < 1 h	2	2	2	2	2	2	2
	Heating, > 1 h	3						
	Cooling to 0°C	4						
	Cooling, < 0°C	5						
**6**	**Workup and purification**							
	None	0						
	Cooling to room temperature	0						
	Adding solvent	0						
	Simple filtration	0						
	Removal of solvent with bp < 150°C	0						
	Crystallization and filtration	1						
	Removal of solvent with bp > 150°C	2						
	Solid phase extraction	2						
	Distillation	3						
	Sublimation	3						
	Liquid-liquid extraction (If applicable, the process includes drying of solvent with desiccant and filtration of desiccant.)	3						
	Classical chromatography	10						
		**Sum penalty points**	5	10	10	2	7	7
		**EcoScale**	95	90	90	98	93	93
